# Organoruthenium Complexes with Benzo-Fused Pyrithiones Overcome Platinum Resistance in Ovarian Cancer Cells

**DOI:** 10.3390/cancers13102493

**Published:** 2021-05-20

**Authors:** Jerneja Kladnik, James P. C. Coverdale, Jakob Kljun, Hilke Burmeister, Petra Lippman, Francesca G. Ellis, Alan M. Jones, Ingo Ott, Isolda Romero-Canelón, Iztok Turel

**Affiliations:** 1 Faculty of Chemistry and Chemical Technology, University of Ljubljana, 1000 Ljubljana, Slovenia; jerneja.kladnik@fkkt.uni-lj.si (J.K.); jakob.kljun@fkkt.uni-lj.si (J.K.); 2 School of Pharmacy, Institute of Clinical Sciences, University of Birmingham, Birmingham B15 2TT, UK; j.coverdale.1@bham.ac.uk (J.P.C.C.); FGE818@alumni.bham.ac.uk (F.G.E.); a.m.jones.2@bham.ac.uk (A.M.J.); 3 Institute of Medicinal and Pharmaceutical Chemistry, Technische Universität Braunschweig, 38106 Braunschweig, Germany; h.burmeister@tu-bs.de (H.B.); p.lippmann@tu-bs.de (P.L.); ingo.ott@tu-bs.de (I.O.)

**Keywords:** ruthenium, pyrithione, resistance to chemotherapy, cancer, ovarian, thioredoxin

## Abstract

**Simple Summary:**

Ovarian cancer is the fifth most common cancer in the developing world, with many front-line treatments combining paclitaxel with platinum-based anticancer agents cisplatin/carboplatin. However, increased incidence of platinum resistance demands the development of new chemotherapeutic agents. The aim of our study was to explore a new family of organoruthenium(II) pyrithione complexes for their efficacy towards platinum-resistant ovarian cancer cells. We confirmed that this new class of compounds remain highly potent towards platinum-insensitive cells and appear to work by a mechanism that is not common to platinum agents.

**Abstract:**

Drug resistance to existing anticancer agents is a growing clinical concern, with many first line treatments showing poor efficacy in treatment plans of some cancers. Resistance to platinum agents, such as cisplatin, is particularly prevalent in the treatment of ovarian cancer, one of the most common cancers amongst women in the developing world. Therefore, there is an urgent need to develop next generation of anticancer agents which can overcome resistance to existing therapies. We report a new series of organoruthenium(II) complexes bearing structurally modified pyrithione ligands with extended aromatic scaffold, which overcome platinum and adriamycin resistance in human ovarian cancer cells. The mechanism of action of such complexes appears to be unique from that of cisplatin, involving G_1_ cell cycle arrest without generation of cellular ROS, as is typically associated with similar ruthenium complexes. The complexes inhibit the enzyme thioredoxin reductase (TrxR) in a model system and reduce cell motility towards wound healing. Importantly, this work highlights further development in our understanding of the multi-targeting mechanism of action exhibited by transition metal complexes.

## 1. Introduction

Ovarian cancer (OC), also known as “the silent killer”, belongs to the most lethal gynecologic malignancies [1,2,3,4,5] and is the fifth most common cancer type in women in the developed world [6]. Although the mortality of patients with an early-stage OC is low, diagnosis at this stage is rather poor due to less obvious and non-specific symptoms. However, once diagnosed with the advanced OC, the survival rate decreases immensely, also due to development of chemotherapy resistance [5]. Thus far, five histological subtypes of OC are known, of which epithelial tumors are the most common, accounting for approximately 90% [7]. Several genetic (*BRCA1/2* gene mutations, DNA mismatched repair gene mutations) and epidemiological risk factors (age, obesity, talcum powder use/asbestos exposure) contribute to the development and the progression of OC [5,8].

Standard OC treatment includes surgery followed by chemotherapy, usually with platinum- (cisplatin, carboplatin) in combination with taxane-based compounds (paclitaxel, docetaxel). As OC is usually discovered at an advanced stage, complete surgical debulking is necessary to maximize the efficacy of subsequent chemotherapy and minimize the likelihood of disease recurrence. Nevertheless, the recurrence rate remains high and is often associated with the development of platinum resistance [7,9]. Intrinsic or acquired multidrug resistance (MDR) to chemotherapeutic agents in patients with advanced OC is responsible for almost 90% of deaths [10]. Cancer cells are capable of developing multiple MDR mechanisms, such as increased xenobiotic metabolism by phase I and II drug-metabolizing enzymes (e.g., cytochromes and glutathione-S-transferase), elevated drug efflux (e.g., ATP-binding cassette proteins such as P-glycoprotein (P-gp)), gene mutations (e.g., very common are tumor suppressor gene *TP53* and *BCR-ABL* gene mutations), elevated production of growth factors (e.g., interleukins, protein kinases, extracellular matrix proteins), and enhanced DNA repair capacity [11]. In OC, overexpression of the efflux protein P-gp is frequently reported, which enables chemotherapeutic agents to be excreted out of the cell, consequently preventing drugs used from interacting with their targets and causing cancer cell death. In addition, OC stem cells, non-coding RNA, increased autophagy, and tumor heterogeneity also contribute to MDR [10].

Platinum drugs are obviously not efficient enough to combat this treacherous disease alone. In response, research groups are now exploring new metal complex architectures based on chemically similar neighboring transition metals, including iridium [12], osmium [13,14,15], and rhodium [16,17]. Besides, complexes based on ruthenium gained significant attention in recent years. Pre-clinical studies and clinical trials of key ruthenium(III) complexes, KP1019 ((IndH)[*trans*-RuCl_4_(Ind)_2_], Ind = indazole), its sodium salt KP1339 (Na[*trans*-RuCl_4_(Ind)_2_]) [18], and NAMI-A ((ImH)[*trans*-RuCl_4_(dmso-S)(Im)], Im = imidazole) [19,20] demonstrated the potential ruthenium compounds offer in the fight against platinum resistance, inspiring the development of future generations of ruthenium-based therapies [21,22,23,24]. Importantly, the in-cell mechanism of action of many Ru(II) or Ru(III) complexes is not solely, or in some cases not at all, dependent on DNA binding interactions, unlike platinum therapies. In fact, many ruthenium complexes were shown to be multitargeting, for instance, by perturbing cellular redox homeostasis, targeting subcellular organelles [25], or disrupting the processes of cell invasion and migration [26]. 

**Figure 1:**
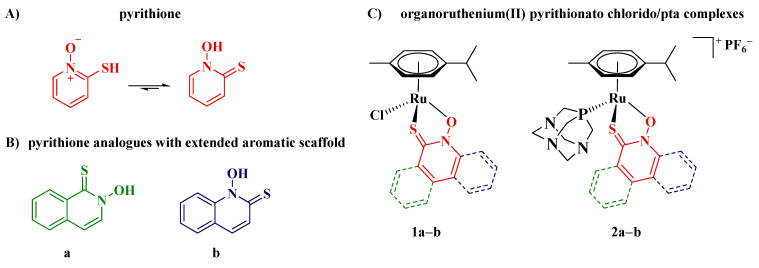
(**A**) Pyrithione and its tautomeric forms. (**B**) Pyrithione analogues with extended aromaticity **a**–**b** and (**C**) their organoruthenium(II) chlorido **1a**–**b** and pta (1,3,5-triaza-7-phosphaadamantane) complexes **2a**–**b**.

In the preclinical stage, some ruthenium complexes were also tested on various OC cell lines. Some ruthenium(III) complexes were evaluated in vitro on cisplatin-sensitive and -resistant rat ovarian tumors [27]. Furthermore, organoruthenium(II) complexes with *N,N-* [22,28,29,30,31], *O,O*- [32,33,34,35,36], *N,S-* [37], and *S-*ligands [38] were extensively studied on OC cell lines. Due to the emergence of new therapeutics for the OC treatment, the aim of this study was to thoroughly investigate organoruthenium(II) complexes (**1a**–**b** and **2a**–**b**, Figure 1C) with isostructural *O,S-*pyrithione type ligands with extended quinoline and isoquinoline scaffolds, namely 2-hydroxyisoquinoline-1(2*H*)-thione and 1-hydroxyquinoline-2-(1*H*)-thione (**a** and **b**, respectively; Figure 1B), for their anticancer properties for this cancer type. Pyrithione (Figure 1A) is a common name for the organic compound that tautomerizes as either 1-hydroxypyridine-2(1*H*)-thione (predominant form) or 2-mercaptopyridine *N*-oxide and has high affinity to most transition metal ions via oxygen and sulphur atoms [39]. In recent years, the Turel group began to focus on the study of organoruthenium(II) complexes with pyrithione and its analogues for their biological properties, mainly anticancer [40,41,42] and, to some extent, antineurodegenerative [43,44]. In this study, we show that condensation of the benzene ring to the pyrithione scaffold increases the activity of complexes towards various ovarian cancer cell lines, including resistant ones. After screening of prepared compounds for their antiproliferative properties against various cancer cell lines, compound **1a** (Figure 1C) was selected for further comprehensive investigation of the mechanisms of action at the cellular level against ovarian cancer cell line A2780, including thioredoxin reductase inhibition, induction of reactive oxygen species, evaluation of mitochondrial function, cell cycle analysis, induction of apoptosis, wound healing assay, and colony formation assay. 

## 2. Materials and Methods

### 2.1. Chemicals

Starting materials and other reagents as well as the solvents for the synthesis of ligands **a**–**b** and complexes **1a**–**b** and **2a**–**b** were purchased from commercial suppliers (Fluorochem, Hadfield, UK; Strem Chemicals, Inc., Newburyport, MA, USA; Merck, Darmstadt, Germany; Honeywell, Muskegon, MI, USA) and used as received, except from CHCl_3_ used for *N*-oxidation, which was dried over Na_2_SO_4_ prior to use. Pta ligand (1,3,5-triaza-7-phosphaadamantane) was prepared as reported [45]. The progress of the reaction was followed by pre-coated TLC sheets ALUGRAM^®^ SIL G/UV254 (Macherey-Nagel, Düren, Germany) under UV light, and column chromatography was carried out on Merck Silica gel 60 (35–70 µm) as stationary phase. 

RPMI-1640 culture medium, penicillin/streptomycin concentrate, glutamine (2 mM solution), trypsin-EDTA, phosphate-buffered saline (PBS), hydrogen peroxide, propidium iodide (PI), RNAse, 2′,7′–dichlorofluorescein diacetate (DCFH-DA), thiazolyl blue tetrazolium bromide (MTT), dimethyl sulfoxide (DMSO), RNAse, Annexin-V-FITC, and tert-butyl hydroperoxide (TBHP) used in biological experiments were purchased from Sigma Aldrich (St. Louis, MO, USA). The 96-well plates assays were carried out using a FLUOStar Omega microplate reader. Microscopy images were obtained using a EVOS PL system. Flow cytometry was carried out using a Beckman Coulter Cytoflex.

### 2.2. Physicochemical Characterization

^1^H and ^31^P NMR spectra were obtained with Bruker Avance III 500 spectrometer at room temperature at 500 MHz and 202 MHz, respectively. Chemical shifts (*δ*) are reported in ppm. ^1^H NMR spectra are referenced to residual peaks of NMR solvent CD_2_Cl_2_ or D_2_O at 5.32 (referenced against the central line of triplet) or 4.79 ppm, respectively, and chemical shifts of ^31^P NMR spectra relative to external standard. The multiplicities are abbreviated as s = singlet, d = doublet, dd = doublet of the doublets, sept = septet, and m = multiplet. Coupling constants (*J*) are given in Hz. MestReNova version 11.0.4 was used for NMR data processing. Infrared spectra were recorded with Bruker FTIR Alpha Platinum ATR spectrometer. High resolution mass spectra (HRMS) were carried out on an Agilent 6224 Accurate Mass TOF LC/MS instrument. Elemental analyses were obtained on a Perkin-Elmer 2400 II instrument (C, H, N). UV–Vis spectra of complexes were obtained on PerkinElmer LAMBDA 750 UV/Vis/near-IR spectrophotometer and UV–Vis stability spectra on UV–Vis spectrophotometer UV-2600 Shimadzu. X-ray diffraction data were collected on an Oxford Diffraction SuperNova diffractometer with Mo/Cu microfocus X-ray source (K_α_ radiation, λ_Mo_ = 0.71073 Å, λ_Cu_ = 1.54184 Å) with mirror optics and an Atlas detector at 150(2) K. The structures were solved in Olex^2^ graphical user interface [46] by direct methods implemented in SHELXT and refined by a full-matrix least-squares procedure based on F^2^ using SHELXL [47]. All non-hydrogen atoms were refined anisotropically. The hydrogen atoms were placed at calculated positions and treated using appropriate riding models. The crystal structures were submitted to the CCDC and were allocated the deposition numbers 2065161–2 065162 for ligand **a** and complex **2b**, respectively. Cyclic voltammetry (CV) was performed using an Autolab PGSTAT100N potentiostat, processed with Nova 2.0 software, cleaned with Microsoft Excel, and graphs were created using SigmaPlot 13.0. Measurements for complexes **1a** and **2a** and ligand **a** (1.0 mM, CH_3_CN containing tetrabutylammonium hexafluorophosphate (0.1 M) as supporting electrolyte) were carried out using degassed solutions under nitrogen, and voltammograms were scanned from +2.0 V to –2.0 V. Alternatively, voltammograms were scanned from +0.8 V to –0.8 V to mimic biological redox potentials. In a typical electrochemical experimental set up, a three-electrode system was used: a glassy carbon electrode as the working electrode, Ag/AgCl as the reference electrode ((+7 mV vs. Fc^+^/Fc) + 205 mV vs. N.H.E.), and platinum wire as the counter electrode. For each electrode, CV was performed at a ν = 100 mVs^–1^.

### 2.3. Syntheses of Ligands and Complexes

Ligands **a**–**b** as well as organoruthenium(II) chlorido complexes **1a**–**b** were resynthesized according to published protocols [44,48], while organoruthenium(II) pta complexes **2a**–**b** were newly prepared following our published protocol [41] as described below (general synthesis pathways presented in Supplementary Materials, Figure S1) and physiochemically characterized (^1^H NMR spectra can be found in Supplementary Materials, Figures S13 and S14).

Appropriate chlorido complex **1a**–**b** (50 mg, 0.112 mmol, 1 mol. equiv.) was dissolved in dichloromethane (30 mL) and finely ground pta ligand (0.168 mmol, 1.5 mol. equivalents) as well as NH_4_PF_6_ (0.168 mmol, 1.5 mol. equivalents) were added and stirred in the dark for 48 h at ambient temperature. After the reaction was completed, the solvent was partly evaporated and filtered through a pad of Celite to remove side product NH_4_Cl and excess of NH_4_PF_6_ and pta. Then, the mother liquor was concentrated on a rotary evaporator to approximately 2 mL. Cold diethyl ether was added to the oily residue, which resulted in precipitation of orange solid, which was washed with diethyl ether and dried at 45 °C overnight.

[η^6^-*p*-Cymene)Ru(II)(2-hydroxyisoquinoline-1(2*H*)-thionato)pta]PF_6_ (**2a**). Yield: 65%. ^1^H NMR (500 MHz, (CD_2_Cl_2_)): *δ* = 8.62–8.58 (m, 1H, Ar–*H* a), 7.85–7.80 (m, 2H, Ar–*H* a), 7.80–7.72 (m, 2H, Ar–*H* a), 7.33 (d, 1H, *J* = 7.3 Hz, Ar–*H* a), 5.98 (d, 1H, *J* = 6.1 Hz, Ar–*H* cym), 5.90 (d, 1H, *J* = 6.1 Hz, Ar–*H* cym), 5.72 (d, 1H, *J* = 6.1 Hz, Ar–*H* cym), 5.59 (d, 1H, *J* = 6.1 Hz, Ar–*H* cym), 4.44 (s, 6H, *H*–pta), 4.13 (dd, 3H, *J* = 15.1, 3.6 Hz, *H*–pta), 3.98 (dd, 3H, *J* = 15.1, 3.6 Hz, *H*–pta), 2.66 (sept, 1H, *J* = 6.9 Hz, Ar–C*H*(CH_3_)_2_ cym), 2.16 (s, 3H, Ar–C*H*_3_ cym), 1.28 (d, 3H, *J* = 6.9 Hz, Ar–CH(C*H*_3_)_2_ cym), 1.25 (d, 3H, *J* = 6.9 Hz, Ar–CH(C*H*_3_)_2_ cym) ppm. ^31^P NMR (202 MHz, CD_2_Cl_2_): *δ* = –31.86 (*P*–pta), –144.31 (sept, *J*_PF_ = 712 Hz, *P*F_6_) ppm. IR selected bands (cm^–1^, ATR): 2971, 974, 947, 836, 801, 742, 670, 580, 557, 480. UV-Vis (*λ* (nm) (*ε* (L mol^−1^ cm^−1^)) *c* = 5 × 10^−5^ M, MeOH): 315 (12268), 393 (4822). ESI-HRMS (CH_3_CN) *m/z* for [M–PF_6_]^+^ (found (calcd)): 569.1078 (569.1078). Anal. Calcd for C_25_H_32_F_6_N_4_OP_2_RuS: C, 42.08; H, 4.52; N, 7.85. Found: C, 42.23; H, 4.52; N, 7.49.

[(η^6^-*p*-Cymene)Ru(II)(1-hydroxyquinoline-2-(1*H*)-thionato)pta]PF_6_ (**2b**). Yield: 56%. ^1^H NMR (500 MHz, (CD_2_Cl_2_)): *δ* = 8.29 (d, 1H, *J* = 8.6 Hz, Ar–*H* b), 7.85–7.78 (m, 2H, Ar–*H* b), 7.69 (d, 1H, *J* = 8.9 Hz, Ar–*H* b), 7.61–7.56 (m, 1H, Ar–*H* b), 7.47 (d, 1H, *J* = 8.9 Hz, Ar–*H* b), 6.02 (d, 1H, *J* = 6.1 Hz, Ar–*H* cym), 5.95 (d, 1H, *J* = 6.1 Hz, Ar–*H* cym), 5.73 (d, 1H, *J* = 6.1 Hz, Ar–*H* cym), 5.58 (d, 1H, *J* = 6.1 Hz, Ar–*H* cym), 4.43 (s, 6H, *H*–pta), 4.10 (dd, 3H, *J* = 15.1, 3.5 Hz, *H*–pta), 3.98 (dd, 3H, *J* = 15.1, 3.5 Hz, *H*–pta), 2.66 (sept, 1H, *J* = 6.9 Hz, Ar–C*H*(CH_3_)_2_ cym), 2.17 (s, 3H, Ar–C*H*_3_ cym), 1.31 (d, 3H, *J* = 6.9 Hz, Ar–CH(C*H*_3_)_2_ cym), 1.25 (d, 3H, *J* = 6.9 Hz, Ar–CH(C*H*_3_)_2_ cym) ppm. ^31^P NMR (202 MHz, CD_2_Cl_2_): *δ* = –31.73 (*P*–pta), –144.29 (sept, *J*_PF_ = 712 Hz, *P*F_6_) ppm. IR selected bands (cm^–1^, ATR): 2946, 1014, 979, 946, 830, 808, 739, 578, 556, 451. UV-Vis (*λ* (nm) (*ε* (L mol^−1^ cm^−1^)) *c* = 5 × 10^−5^ M, MeOH): 292 (20472), 357sh (5604), 412 (4322). ESI-HRMS (CH_3_CN) *m/z* for [M–PF_6_]^+^ (found (calcd)): 569.1083 (569.1078). Anal. Calcd for C_25_H_32_F_6_N_4_OP_2_RuS: C, 42.08; H, 4.52; N, 7.85. Found: C, 41.63; H, 4.79; N, 7.87.

### 2.4. Aqueous Stability

For NMR stability, approximately 4 mg of compound was first dissolved in (CD_3_)_2_SO to which D_2_O was added to obtain 5% (CD_3_)_2_SO/D_2_O final solution (600 µL). In case of the stability experiments with NaCl, NaCl was first dissolved in D_2_O, and a latter solution was consequently added to (CD_3_)_2_SO solution with the complex to obtain 5% (CD_3_)_2_SO/D_2_O final solution containing 140 mM NaCl (600 µL). ^1^H NMR spectra were recorded immediately after the preparation of the solutions and later at the selected timepoints.

UV-Vis stability was studied over 24 h using UV-visible spectroscopy in biologically relevant matrixes: a) PBS, b) RPMI-1640, c) fully prepared RPMI-1640 which included the addition of 10% *v/v* fetal calf serum and 1% *v/v* pen/strep antibiotics, and d) human blood plasma. DMSO stock solutions of tested complexes were prepared and further diluted in the mentioned matrixes. UV-Vis spectra were obtained immediately after the preparation of solutions and after 24 h between 250 and 900 nm using single beam scans with background correction. Between these two measurement timepoints, samples were kept in sealed cuvettes at 37 °C.

### 2.5. Human Cancer Cell Culture

Human cells lines (A2780, SW626, SKOV3, A2780Cis, A2780ADR, A549, HEPG2, OE19, PC3, HCT116) were purchased from the European Collection of Cell Cultures (ECACC) and tested at regular intervals to confirm mycoplasma free status. Cells were grown as adherent monolayers using RPMI-1640 culture medium supplemented with 10% *v/v* fetal calf serum, 1% *v/v* penicillin/streptomycin antibiotics, and 1% *v/v* 2 mM glutamine. Cells were maintained using 25 or 75 cm^2^ flasks at 37 °C in a humidified atmosphere containing 5% CO_2_ and passaged at regular intervals using trypsin-EDTA upon reaching 80–90% confluence.

### 2.6. Determination of Antiproliferative Activity

Using flat-bottom 96-well plates, 5 × 10^3^ cells were seeded per well in drug-free media and incubated at 37 °C K for 48 h. Prior to the treatment of cells, a stock solution of a test compound was prepared using a 1:1 mixture of cell culture medium and saline (0.9%) and a starting concentration of 5% *v/v* DMSO to aid complex solubility. Serial dilutions of the stock solution were prepared to ensure the final working DMSO concentration did not exceed 0.5% *v/v*. Cells were treated with six concentrations of test complex between 200 and 0.01 µM for 24 h at 37 °C, then the supernatant solution was aspirated, cells were washed with PBS, and cells were re-incubated in fresh (drug-free) media. After 72 h further incubation at 37 °C (so-called “recovery time”), cell viability was determined using the MTT assay (4 h dark exposure to MTT reagent). After this time, dye was solubilized in DMSO, and absorbance measurements were obtained using a microplate reader. Cell viabilities and respective IC_50_ concentrations (the concentration of tested complex which caused 50% growth inhibition) were calculated relative to untreated controls as part of duplicate of triplicate experiments in two independent experiments. 

### 2.7. Induction of Apoptosis

Briefly, 2 × 10^5^ A2780 human ovarian cancer cells were seeded in flat-bottom 6-well plates and incubated at 37 °C for 24 h in a CO_2_ humidified atmosphere. Cells were treated with 1 µM concentrations of complex **1a** in cell culture media (DMSO not exceeding 0.5% *v/v*), prepared as described previously. After 24 h incubation, the supernatant solution was removed by aspiration, and adherent cells were washed with PBS and collected using trypsin. After quenching of trypsin activity with culture media (containing FCS), cells were resuspended as a single cell solution in buffer containing propidium iodide (PI, Ex. 560 nm, Em. 595 nm) and Annexin-V-FITC conjugate (Ex. 485 nm, Em. 535 nm). Separately, negative (untreated) and positive (1 µg/mL staurosporine) control samples were prepared. No cellular fixation protocols were employed to circumvent non-specific binding of Annexin-V-FITC. Samples were analyzed using flow cytometry with gating determined using positive and negative control samples. The experiment was carried out as three independent biological replicates. 

### 2.8. Cell Cycle Analysis

Briefly, 1 × 10^6^ A2780 human ovarian cancer cells were seeded using flat-bottom 6-well plates and incubated at 37 °C for 24 h. Cells were treated with 1 µM concentrations of complex **1a** in cell culture media (DMSO not exceeding 0.5% *v*/*v*), prepared as described previously. After 24 h incubation at 37 °C, the supernatant solution was removed by aspiration, and adherent cells were washed with PBS and collected using trypsin. Negative (untreated) control samples were also prepared. After quenching of trypsin activity with culture media (containing FCS), cells were resuspended in ice-cold ethanol for 2 h. Ethanol was removed by centrifugation, and cell pellets were suspended in fixation buffer containing propidium iodide (PI, Ex. 560 nm, Em. 595 nm) and RNAse A. Cells were analyzed using flow cytometry, and data were processed using Flowjo for Windows. Experiments were carried out as three independent biological replicates.

### 2.9. Wound Healing Assay

Briefly, 24-well plates were seeded using 1 × 10^4^ A2780 human ovarian cancer cells per well and incubated at 37 °C for 24 h. After this time, a sterile pipette tip was used to create two “wounds” per well, and cells were treated with 1 µM complex **1a** for 24 h. The supernatant solution was aspirated, and cells were washed with PBS and stained using crystal violet solution (10% ethanol). After staining, excess stain was removed using PBS, and cells were visualized using a transmission microscope using 4× optical zoom. Values were calculated out of 36 random measurements of three wounds (12 measurements in each wound).

### 2.10. Colony Formation Assay

A2780 cancer cells were seeded in P100 flasks at a density of 1 × 10^3^ and incubated for 24 h at 37 °C before administration of 1 µM concentration of complex **1a** in culture medium (DMSO not exceeding 0.5% *v*/*v*), prepared as described previously. After this time, cells were washed and allowed a further seven days of growth at 37 °C in fresh media. Then, colonies were counted. This experiment was carried out in duplicate with independent triplicate experiments.

### 2.11. Induction of Reactive Oxygen Species (ROS)

Briefly, 1 × 10^4^ A2780 human ovarian cancer cells were seeded using 96-well black plates and incubated at 37 °C for 24 h in a humidified atmosphere. Cells were treated with 1 µM concentrations of complex **1a** in cell culture media (DMSO not exceeding 0.5% *v*/*v*), prepared as described previously. After 24 h incubation at 37 °C, the supernatant solution was removed by aspiration, adherent cells washed with PBS, and to each well was added 100 µL of 50 µM of 2′,7′–dichlorofluorescein diacetate (DCFH-DA), after which time, plates were incubated in the dark for 2 h. After staining, cells were washed thoroughly with PBS to remove excess stain before adding ROS inducers to positive control samples (1 mM hydrogen peroxide or 0.5 mM tert-butyl hydroperoxide) for 2 h in the dark. Fluorescence readings were obtained using a microplate reader (λ_Ex_ = 485 nm, λ_Em_ = 530 nm). Additional control samples included negative (untreated) controls, controls only treated with metal complexes (to eliminate auto-fluorescence), and complex-treated cells with ROS inducers. Data were obtained as biological triplicates. Generation of ROS was also investigated using fluorescence microscopy. Samples were prepared as described for quantification above with the following modification: cells were seeded using 8-well microscopy chambers using 5 × 10^4^ cells per well. Measurements were obtained using an EVOS FL microscope.

### 2.12. Evaluation of Mitochondrial Function

Briefly, 5 × 10^3^ A2780 human ovarian cancer cells were seeded in 8-well microscopy chambers and incubated at 37 °C for 24 h. Cells were treated with 1 µM concentrations of complex **1a** in cell culture media (DMSO not exceeding 0.5% *v*/*v*), prepared as described previously. After 24 h incubation at 37 °C, the supernatant solution was removed by aspiration, and adherent cells washed with PBS and stained using DAPI/Rhodamine 123 (Rh-123) in buffer for 1 h. This experiment included negative (untreated) controls. Measurements were obtained as biological triplicates using an EVOS FL microscope.

### 2.13. Thioredoxin Reductase Assay

Assay was carried out as in previously reported detailed protocols [49,50]. In short, commercially available rat liver TrxR (Sigma-Aldrich, St. Louis, MO, USA) was exposed to graded concentrations of the test compounds over 75 min. After this exposure period, the reduction of 5,5′-dithiobis (2-nitrobenzoic acid) mediated by NADPH and TrxR to 5-thio-2-nitrobenzoic acid was monitored photometrically at 405 nm. The IC_50_ values represent the concentrations, which were required to reduce the enzyme catalyzed turnover by 50%.

### 2.14. Cellular Accumulation of Ruthenium

Briefly, 1 × 10^6^ cells were seeded using P100 plates and incubated at 37 °C for 24 h. Cells were treated with IC_50_ concentrations of complex **1a** or **2a** in cell culture media (DMSO not exceeding 0.5% *v*/*v*), prepared as described previously. After 24 h incubation at 37 °C, the supernatant solution was removed by aspiration, and adherent cells were washed with PBS and harvested using trypsin. Trypsin activity was quenched using culture medium (containing FCS), cells were counted, and cell pellets were obtained by centrifugation. Cell pellets were re-washed with PBS to remove excess culture medium and re-centrifuged to afford the final cell pellet for analysis. The protein content of the cell pellets was determined by the Bradford method, and the ruthenium levels were measured using a high-resolution continuum source atomic absorption spectrometer (HRCS-AAS; contrAA 700 AnalytikJena) according to previously reported protocols [50,51].

### 2.15. Statistical Analysis

In all cases, independent two-sample t-tests with unequal variances, Welch’s tests, were carried out to establish statistical significance of the variations (*p* < 0.01 for **, and *p* < 0.05 for *).

## 3. Results and Discussion

### 3.1. Syntheses and Crystal Structures of the Ligands and Complexes

Compounds that were evaluated for their anticancer properties are presented in Figure 1 (B—ligands, C—complexes). Isostructural ligands **a** and **b** (Figure 1B) are isoquinoline and quinoline-derived pyrithione analogues, prepared in a two-step synthesis, involving *N*-oxidation followed by thiolation (Figure S1A) [44,48]. With ligands in hand organoruthenium(II) chlorido complexes **1a**–**b** (Figure 1C) were prepared by stirring ruthenium precursor [Ru(*p*-cymene)Cl_2_]_2_, selected pyrithionato-ligand **a** or **b,** and a base sodium methoxide in dichloromethane (Figure S1B). After the purification of reaction mixture by column chromatography, complexes **1a**–**b** were isolated upon precipitation from DCM/*n*-heptane [44]. Reactions to afford organoruthenium(II) pta complexes **2a**–**b** (Figure 1C; Figure S1B) were carried out using chlorido complexes **1a** or **1b**, respectively, which were stirred with ammonium hexafluorophosphate (NH_4_PF_6_) and ground pta ligand, both of which were present in excess. The role of NH_4_PF_6_ was to facilitate the abstraction of the chlorido ligand from ruthenium (II) species and the subsequent binding of the phosphine ligand pta. After the reaction was completed, the excesses of NH_4_PF_6_ and pta as well as the insoluble byproduct NH_4_Cl were filtered off through a pad of Celite, and the mother liquor was concentrated on a rotary evaporator. The addition of cold diethyl ether resulted in the precipitation of the yellow solid complexes, which were isolated by filtration.

**Figure 2:**
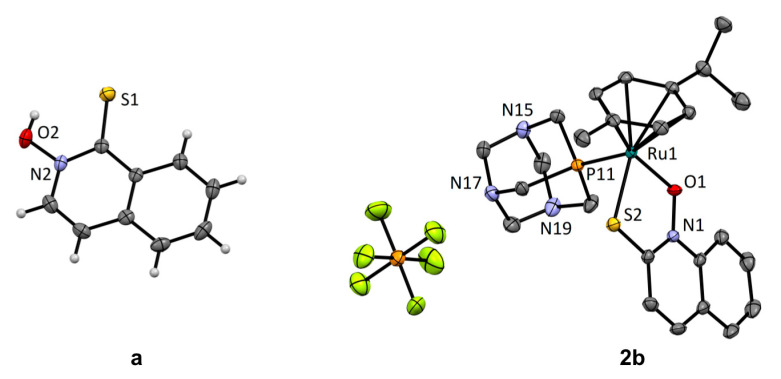
Crystal structures of ligand **a** and pta complex **2b**. Thermal ellipsoids are drawn at 35% probability level.

Crystal structures of chlorido complexes **1a**–**b** were already reported [44]. However, we additionally obtained suitable crystals of ligand **a** and pta complex **2b** for single crystal X-ray diffraction (Figure S2). Ligand **a** crystallized in the thione form (Figure 2), similar to all other previously reported pyrithione analogues [41]. The packing of molecules was characterized by the formation of dimers through O–H···S hydrogen bonds of the thiohydroxamate functional groups and π-stacking interactions of the isoquinoline rings with the distance between planes defined by the 10 ring atoms of 3.403 Å (Figure S3). The crystal structure of pta complex **2b** showed pseudooctahedral geometry of the ruthenium (II) coordination sphere with the bidentately bound ligand **b** forming a cationic structure (Figure 2, Table S1).

### 3.2. NMR Stability in Solution

NMR stability studies were carried out for chlorido **1a**–**b** and pta **2a**–**b** complexes to follow possible structural changes in simplified aqueous solutions of 5% (CD_3_)_2_SO/D_2_O in the presence or the absence of 140 mM NaCl (Figures S4–S11). The addition of the salt mimics high concentrations of extracellular fluids of the body, while studies in water allow observation of possible hydrolysis events. It is reported that Ru–Cl bond in structurally similar complexes is prone to undergo substitution with the neutral water molecules and form positively charged Ru-OH_2_ species which can further interact with biological targets [52]. Moreover, in our previous studies, we found that the behavior of various organoruthenium complexes in solutions depends on the type of the ligand and affects interactions with biomolecules and, consequently, their biological activity [41,53,54].

Interestingly, ruthenium pyrithione chlorido complexes **1a** and **1b** were observed to remain stable in both water and saline solutions, with only minor release of *p*-cymene ligand (around 2%) from the metal observed after 24 h (Figures S4–S7), and the chlorido complex remained the major species in solution. In contrast, ruthenium pta complexes **2a** and **2b** appeared less stable in both water and saline (Figures S8–S11), and after 4 days, the free *p*-cymene ligand dominated over those of the coordinated pta complex. Additionally, new peaks were identified (4.4–4.2 ppm) which can be likely attributed to an uncoordinated pta derivate (pta oxide, 1,3,5-triaza-7-phosphaadamantane-7-oxide) as previously described for similar pta complexes [41]. However, we cannot unambiguously confirm the presence of pta oxide, as these new peaks do not precisely coincide with a referenced spectrum of free pta oxide. 

### 3.3. Antiproliferative Activity

We first established the anticancer activities of (iso)quinoline-derived pyrithione ligands **a**–**b**, their organoruthenium(II) chlorido **1a**–**b,** and pta complexes **2a**–**b** towards six human cancer cell lines—A2780 (ovarian), A549 (lung), HCT116 (colorectal), OE19 (oesophageal), HEPG2 (hepatocellular), and PC3 (prostate)—compared to the established platinum anticancer agent, cisplatin. Our results demonstrate that organoruthenium(II) chlorido complexes **1a** and **1b** showed moderate antiproliferative activity across a panel of human cancer cell lines (Table 1), however, they were particularly active towards ovarian cancer cells (IC_50_ 1.0–2.2 µM). Ligands **a** and **b** from which the complexes were derived also exhibited modest antiproliferative activities towards all tested cancer cell lines (Table 1), however, to a lesser extent than their chlorido complexes. The ruthenium *p*-cymene precursor [Ru(*p*-cymene)Cl_2_]_2_ of complexes **1a** and **1b** is known to be inactive towards cancer cells [22,55,56,57,58], and our group determined its activity on A2780 and A549 cells with similar results (Figure S15). Hence, the enhancement of ligand anticancer activity can be attributed to the formation of the new complex. In contrast, pta complexes **2a** and **2b** were found to be inactive towards all cell lines in the concentration range investigated. This is particularly interesting when considering the established mechanism of action (MoA) of some structurally-similar ruthenium and osmium piano stool complexes, for which hydrolysis and/or displacement of the metal-halide bond at the monodentate site is known to be crucial to in-cell activation [59,60]. It appears that the pta moiety disrupts this process, thereby significantly reducing the potency of these complexes compared to their chlorido analogues, **1a** and **1b**. Interestingly, substitution of the chlorido ligand for pta in previously reported methyl-substituted pyrithione complexes did not affect anticancer activities [41], however, our data suggest that the nature of the monodentate ligand is of great importance in the MoA of ruthenium pyrithione complexes bearing an extended aromatic system.

Platinum therapies are widely used in the first line treatment of ovarian cancers, however, resistance is an ongoing clinical concern. Thus, anticancer activities were next determined towards three ovarian epithelial cancer cell lines, A2780, SKOV3, and SW626 (Table 2). Activity trends between all tested compounds were highly similar, with comparable activities observed towards A2780 (Figure S16) and SW626 and slightly lower activities determined towards SKOV3 cancer cells. A2780 ovarian cancer cells are highly sensitive to cisplatin, whereas ectopic expression of the CA125 tumor antigen C-terminal domain in SKOV3 cells decreases their sensitivity to cisplatin [61]. Interestingly, SW626 cancer cells are also thought to be primary ovarian cells, though studies suggested that they might be derived from an ovarian metastasis of a colorectal adenocarcinoma [62].

All ligands and chlorido complexes appeared slightly less active towards SKOV3 cells compared to platinum-sensitive A2780 cells. The observed variations in activity could include a contribution from intrinsic cellular differences between A2780 and SKOV3. These cells differ in proliferation rates, receptor expression, and morphology.

Therefore, we also determined antiproliferative activities of the most promising complexes, **1a** and **1b**, towards a platinum-resistant variant of A2780 (A2780Cis) to provide direct comparison between platinum-sensitive and platinum-resistant cells (Table 2). Strikingly, the activities of **1a** and **1b** were statistically unchanged between these two cell lines, suggesting that these complexes show promise in the fight against platinum resistance. 

In addition to platinum resistance in the clinic, some cancers may acquire resistance to a range of chemotherapeutics, becoming so-called “multi-drug resistant” (MDR) cancers. Thus, the antiproliferative activities of **1a** and **1b** were also determined towards A2780ADR, an Adriamycin-resistant variant of the parent A2780 cell line (Table 2). Both complexes achieved comparably low micromolar IC_50_ concentrations, between 1.0–1.6 µM for the complex **1a** and 2.2–2.8 µM for the complex **1b**. Given the likely importance of the Ru−Cl bond in the mechanism of action and how orientation of either isomer does not inflict steric interference at the monodentate site, it is perhaps not surprising that, overall, **1a** and its structural isomer **1b** exhibited comparable antiproliferative activities across all human cell lines investigated. 

### 3.4. Cellular Accumulation of Ruthenium

Metal complexes may be transported across cell membranes by passive diffusion, transport proteins, or endocytosis [63], where subsequent in-cell activation and anticancer activity may occur [59,64]. In addition, efflux mechanisms should also be considered. In combination, both influx and efflux pathways can contribute to drug resistance [63]. For example, cisplatin-resistant A2780Cis cells exhibit downregulation of a copper uptake transporter protein (CTR1) and upregulation of efflux transporters multi-drug resistance protein 2 (MRP-2), ATPase copper transporting α (ATP7A), and ATPase copper transporting β [65].

Cellular accumulations of ruthenium in cells treated with either active complex **1a** or **2a** were determined after 24 h of incubation with equimolar concentrations (1.0 µM) of metal complex. Ruthenium was found to be internalized by A2780 cancer cells treated with either chlorido **1a** or its analogue pta complex **2a**, with average accumulations of 1.30 nmol (131.4 ng) Ru/mg protein and 1.02 nmol (103.1 ng) Ru/mg protein, respectively (Table 3). Since A2780 cells were incubated with equimolar concentrations of **1a** and **2a**, it is perhaps unsurprising that metal accumulations were found to be highly comparable, suggesting that these structurally similar complexes may share a common mechanism of cellular influx. However, what is striking is how similar levels of internalized complex differed in their anticancer activity between (IC_50_ = 1.0 µM and > 50 µM for **1a** and **2a**, respectively). Thus, while the nature of the monodentate site does not appear to influence accumulation, these data provide further evidence for its importance in the overall mechanism of action.

It was previously demonstrated for structurally similar organoruthenium(II) chlorido complexes that cellular uptake does not corelate with the cytotoxicity of the compounds towards A2780 cells [66]. Interestingly, other organoruthenium(II) pta complexes were reported to be inactive on TS/A adenocarcinoma cancer cells despite their cellular uptake [67], as observed with complex **2a**. 

### 3.5. Interactions in Biological Media

**Figure 3:**
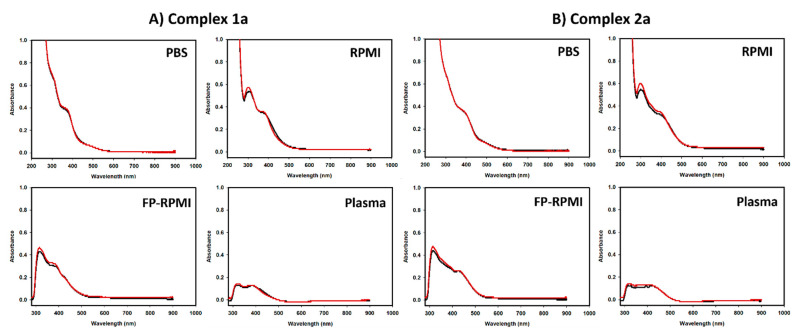
UV-Vis spectra of (**A**) organoruthenium(II) chlorido **1a** and (**B**) pta complex **2a** in PBS, RPMI-1640, FP-RPMI-1640, and human blood plasma recorded immediately after the preparation (black) and after 24 h (red).

NMR stability studies suggested that ruthenium pta complexes **2a**–**b** may undergo greater structural changes in solution (Figures S8–S11) compared to their chlorido analogues **1a**–**b** (Figures S4–S7). To gain further insight into the stability of the compounds in a more complex and biologically relevant environment, the compounds were further studied by UV-visible spectroscopy using (i) phosphate buffer solution PBS, (ii) cell culture medium RPMI-1640, (iii) cell culture medium RPMI-1640 supplemented with 10% (*v*/*v*) fetal calf serum and 1% (*v*/*v*) penicillin/streptomycin antibiotics, and (iv) human blood plasma by UV-Vis spectrometry. As similarly observed in preliminary NMR stability studies, chlorido complexes **1a** and **1b** (Figure 3A and Figure S12A, respectively) remained stable in all four media. While pta complexes **2a** and **2b** retained modest stability in phosphate-buffered saline, as observed in NMR studies, spectral changes were observed in all other media (Figure 3B and Figure S12B, respectively). The observed UV-Vis spectral changes of the pta complexes could relate to a commonality between RPMI-1640 culture medium (in the fetal calf serum supplemented and non-supplemented samples) and human blood plasma. Hence, addition of fetal calf serum to the supplemented media sample was not essential to causing this change. Both RPMI-1640 and human plasma contain significant levels of nucleophilic components (e.g., 1 mM reduced glutathione in RPMI-1640, with similar levels measured in human plasma [68]) which may interfere with coordinated pta. More generally, UV-Vis studies together with NMR stability experiments point to more possible structural changes of investigated pta than chlorido complexes that might contribute to the lack of observed in vitro activity of **2a** and **2b** in the presence of cell culture medium.

### 3.6. Explorations of the Mechanism of Action

We previously reported similar pyrithione ligands which became effective inhibitors of thioredoxin reductase (TrxR) when associated with the architecture of a metal complex [41]. This enzyme (alongside thioredoxin and NADPH) makes up the thioredoxin system, which is involved in processes such as control of the intracellular redox environment, cellular growth, and the regulation of apoptosis [69]. As such, the thioredoxin system is an interesting target in the development of anticancer drugs [70]. In fact, overexpressed thioredoxin in some cancer cells was implicated as a contributor to platinum resistance due to its involvement in the regulation of apoptosis [71]. Similarly, inhibition of thioredoxin reductase by the gold(I) drug auranofin was proposed to enhance cell death in cisplatin-resistant human ovarian cancer cells [72]. 

As complex **1a** retained activity towards both platinum sensitive (A2780) and resistant (A2780Cis) cancer cells, interactions between complex **1a** and thioredoxin reductase were investigated. During the preliminary assay, a concentration of 10 µM complex **1a** caused 94% inhibition of TrxR, with an IC_50_ of 4.1 ± 0.16 µM determined in dose-response experiments. We also observed that ligand **a** (from which complex **1a** was derived) did not inhibit TrxR. Considering that the determined inhibitory concentration of TrxR (4.1 µM) was also comparable with the determined anticancer growth inhibition concentration (1.0 µM), TrxR is a possible subcellular target of **1a** and may contribute to the in vitro mechanism of action. Involvement of TrxR in the MoA of a multi-targeting metal complex presents a possible explanation for the ability of **1a** to overcome platinum resistance in A2780 cancer cells. 

**Figure 4:**
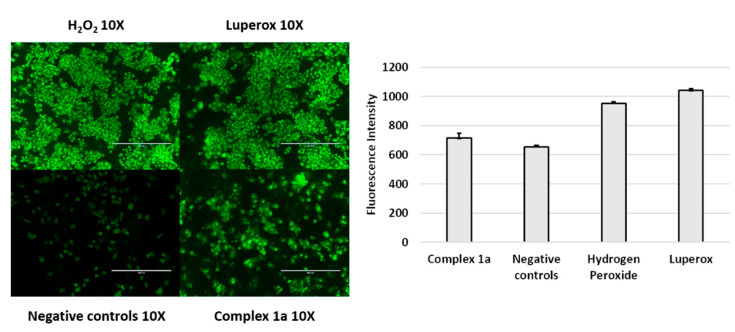
A2780 cancer cells stained with DCFDA. Samples include no treatment (negative control), 24 h exposure at 37 °C to complex **1a** (1.0 µM), as well as 1 h exposure to hydrogen peroxide (H_2_O_2_) and 1 h exposure to Luperox. Fluorescence measured using an EVOS fluorescence microscope (λ_Ex_ = 485 nm, λ_Em_ = 530 nm).

Since complex 1a appears to inhibit thioredoxin reductase activity in model experiments, and TrxR is linked to cellular redox regulation, we explored evidence for this observation in vitro by fluorescence microscopy. While low to moderate concentrations of reactive oxygen species (ROS) are reported to be important for normal functioning of some physiological processes, elevated ROS species can damage proteins, nucleic acids, lipids, and membranes, which may activate either non-physiological (necrotic) or regulated (apoptotic) cell death pathways [73,74]. Many metal complexes are known to cause enhanced ROS formation [75,76,77,78]. We therefore evaluated the induction of reactive oxygen species (ROS) in A2780 cells by complex 1a. However, our results showed that the compound 1a only induced a small increase in intracellular ROS levels (fluorescence intensity ca. 700 fluorescence units) compared to the untreated control (ca. 650 fluorescence units; Figure 4), with measured ROS levels in treated cells only slightly above basal signaling levels in untreated cells. In contrast, positive controls hydrogen peroxide and Luperox displayed significantly elevated fluorescence relative to the untreated control (ca. 950 and 1050 fluorescence units, respectively).

**Figure 5:**
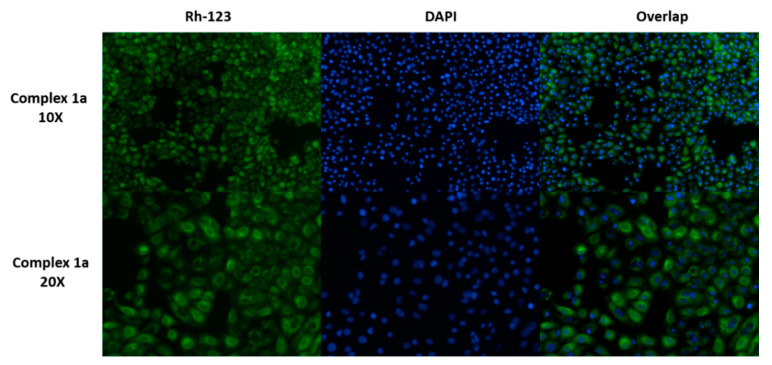
A2780 cancer cells stained with Rhodamine-123 (green) or DAPI (blue) after incubation of cells with complex **1a** (1.0 µM) for 24 h at 37 °C. Fluorescence measured using a fluorescence microscope using Rh-123 (λ_Ex_ = 511 nm; λ_Em_ = 534 nm) and DAPI (λ_Ex_ = 340 nm; λ_Em_ = 488 nm).

Reactive oxygen species are the byproduct of oxidative phosphorylation, and enhanced ROS concentrations are often correlated with mitochondrial dysfunction [79]. Since levels of ROS were relatively unchanged by treatment of A2780 cells with **1a**, mitochondrial function in treated cells was also evaluated to provide further evidence that ROS are unlikely to play a major role in the in-cell mechanism of complex **1a** (Figure 5). In both untreated controls and treated cells, blue fluorescence (DAPI) suggested that cells did not exhibit significant nuclear morphology changes, consistent with previous experiments. Similarly, green fluorescence (Rhodamine-123) showed that mitochondrial function was unaffected by treatment with **1a** compared to the untreated control (Figure S17) under the conditions investigated.

**Figure 6:**
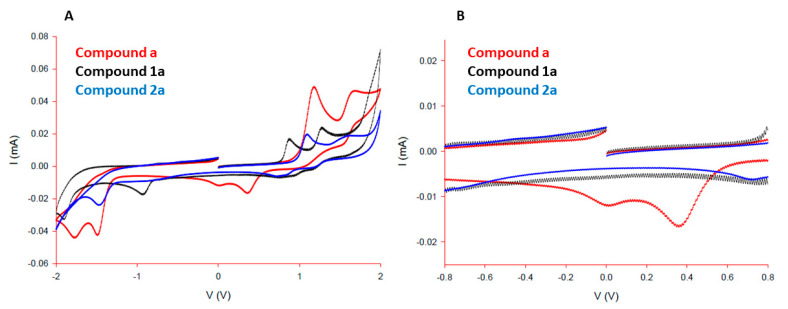
Cyclic voltammograms for the compounds **a**, **1a** and **2a**: (**A**) full scan and (**B**) biologically relevant scan region.

Further evidence of the lack of redox involvement of complex **1a** was provided by cyclic voltammetry studies, including ligand **a**, and analogous pta complex **2a**. While all three compounds are redox active between –2.0 V to +2.0 V (Figure 6A), only uncoordinated ligand **a** exhibited redox activity between the biologically-relevant range of –0.8 V and +0.8 V (Figure 6B). Thus, while ruthenium pyrithione complexes **1a** and **2a** are unlikely to be involved in cellular redox processes, it is possible that cellular degradation pathways may mediate release of free ligand **a**, which may account for the observations of slightly elevated ROS levels compared to the untreated control (Figure 4). Such degradation pathways were previously identified for structurally similar osmium-arene complexes utilizing combination studies of ICP-MS and X-ray fluorescence microscopy, involving endosomal degradation and glutathione [80]. 

Upon excluding ROS and mitochondrial targeting from the likely major mechanism of complex **1a** in cells, a cell cycle analysis of A2780 cancer cells treated with compound **1a** was determined (Table 4). In platinum-sensitive cells, platinum-based therapies exhibit S + G_2_/M-phase cell cycle arrest as a result of binding DNA, thereby preventing DNA synthesis. This results in the activation of p53/p21 pathways, leading to cellular apoptosis [81]. However, in platinum-resistant cells, DNA repair mechanisms such as the nucleotide excision repair systems (NER) can repair platinated DNA adducts, preventing S+G_2_/M cell cycle arrest and promoting platinum resistance [82]. In order to overcome platinum resistance, multi-targeted compounds must identify unique subcellular targets that are not common to platinum drugs. After incubation of A2780 cancer cells with complex **1a**, the cell cycle G_1_ population was significantly increased relative to the untreated control (72.5% compared to 62.5%, respectively). As such, it appears that the mechanism of action of **1a** is not common to platinum-based compounds. It is well established that metal complexes often exhibit multi-targeting mechanisms [24], and we suggest that the compound **1a** is likely to interact with various sub-cellular components other than DNA. 

On detecting significant G_1_ arrest caused by complex **1a**, the induction of apoptosis (programmed cell death) in A2780 cancer cells was investigated to determine whether the mechanism of **1a** was cytotoxic or cytostatic (Table 5). Cells were stained with propidium iodide (PI) and Annexin-V (FITC conjugate; ANN) to identify loss of membrane integrity (PI able to enter cells and bind to DNA) and the translocation of phosphatidylserine to the outer membrane, a trait commonly associated with late-stage apoptosis. A2780 cells treated with complex **1a** only showed partial evidence for apoptotic cell death (8.4% of cell population), with a greater cell population determined as non-viable cells (11.2% of cell population), indicative of a major non-apoptotic pathway in the mechanism of action. Apoptotic cell death pathway is frequently identified with platinum agents, and its activation is mediated by a variety of mechanisms. For example, while platinum-associated apoptosis is typically dependent on the activation of tumor suppressor protein p53, ruthenium complexes were reported to induce ERK1/2-mediated apoptosis via a p53-indpendent pathway [83]. While it appears that this new class of ruthenium complexes does cause slight apoptosis, this level is unlikely to contribute to the main mechanism of action of such complexes towards A2780 cancer cells.

**Figure 7:**
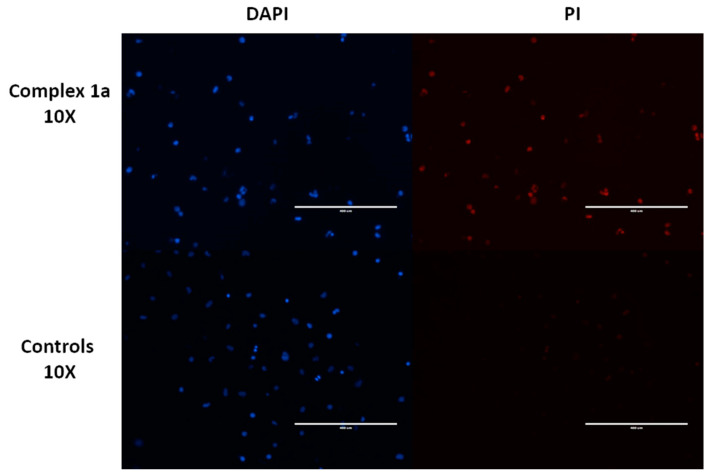
Evaluation of membrane integrity of A2780 cancer cells treated with complex **1a** (1.0 µM) for 24 h at 37 °C compared to the untreated control. Unfixed cells were stained using DAPI (blue) or propidium iodide (red), showing clear disruption of membrane integrity in the presence of complex **1a**, thus allowing PI to enter cells and fluoresce upon binding DNA.

Owing to the large population of non-viable cells after treatment with **1a**, the membrane integrity of exposed cells was evaluated. Cells exhibiting compromised membranes in the absence of apoptotic cell death may indicate the involvement of a necrotic cell death pathway. Interestingly, while cells did not exhibit significant nuclear morphology changes (viable blue DAPI florescence), complex **1a** appeared to disrupt the cell membrane, allowing propidium iodide (PI) to enter cells, causing red florescence upon binding cellular DNA (Figure 7). Further, this finding is consistent with the observation of a greater population of cells in the PI+ quadrant during the investigation of apoptosis (Table 5).

**Figure 8:**
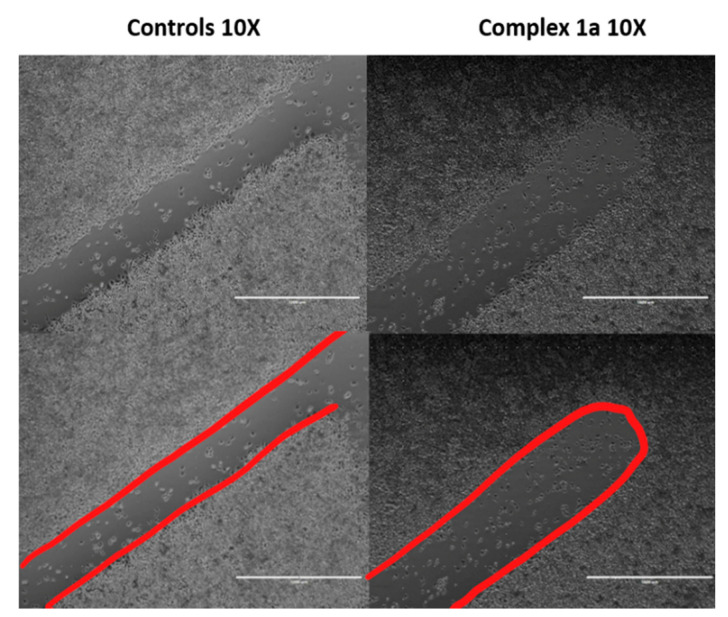
Wound healing assay for A2780 cancer cells after wounding using a pipette tip (average day 0 wound size 1282 ± 59 µm) after 24 h incubation at 37 °C in the presence or the absence (untreated control) of complex **1a** (1.0 µM). Complex **1a** significantly reduces cell motility towards the wound (*p* < 0.05).

The influence of complex **1a** on the migration of A2780 cancer cells was next investigated as part of a wound-healing assay (Figure 8). A wound was created in a monolayer of A2780 cells using the pipette tip (1282 ± 59 µm). Cells treated with complex **1a** were observed and compared to the untreated (negative) control to determine whether the presence of the ruthenium complex would influence wound healing or cell motility. After 24 h exposure to **1a**, the wound healing was significantly hindered (*p* < 0.05) compared to the untreated cell population, exhibiting a wound width of 1035 ± 89 µm (19% reduction) compared to 319 ± 35 µm (75% reduction), respectively. Cells treated with **1a** showed a reduction in motility towards wound healing, which is consistent with previously observed G_1_ arrest. Since such cell cycle arrest extends the growth phase and delays DNA replication and subsequent cell division, these findings suggest that the mechanism of action appears to be cytostatic, as opposed to cytotoxic. In addition, cell colonies treated with complex **1a** for 24 h were found to be significantly smaller and fewer in number (24 ± 5 colonies) compared to untreated colonies (45 ± 3 colonies) after 7 days growth (*p* < 0.01). This result is again consistent with the observation of G_1_ cell cycle arrest and a cytostatic (rather than cytotoxic) mechanism of action. 

## 4. Conclusions

A new series of organoruthenium(II) pyrithione complexes were described, which show promising potency towards ovarian cancer cells. The nature of the monodentate site was confirmed to be crucial in the activation and/or the mechanism of action of these complexes, as substitution of the chlorido moiety for pta rendered the complexes inert under the test conditions of our experiments. This work suggests that the mode of action of **1a** is cytostatic in nature, involving G_1_ cell cycle arrest, the absence of ROS generation of mitochondrial dysfunction, reduced cell motility, reduced cell colony size, and limited induction of apoptosis. Similarly, cells treated with **1a** were found to exhibit damaged cell membranes, in agreement with the increased non-viable cell population determined by flow cytometry as part of apoptosis experiments. Importantly, the mechanism of action appears to differ from the well-established DNA binding mechanism of action of platinum anti-cancer agents, and our findings offer an explanation as to how complex **1a** appears to overcome platinum resistance in ovarian cancer cells. In addition, complex **1a** also inhibits TrxR, enzyme involved in cancer development. Our findings highlight how a multi-targeting mechanism of action offered by such metal complexes can contribute to future development of cancer therapies to combat drug resistance.

## Figures and Tables

**Table 1:** Antiproliferative activities of the prepared compounds towards six human cancer cell lines: A2780 (ovarian), A549 (lung), HCT116 (colorectal), OE19 (oesophageal), HEPG2 (hepatocellular), and PC3 (prostate).

Compound	IC_50_ (µM) [a]					
	A2780	A549	HCT116	OE19	HEPG2	PC3
**a**	8.6 ± 0.2	11.6 ± 0.8	17.5 ± 0.4	12.1 ± 0.4	32.5 ± 0.6	16.5 ± 0.3
**1a**	1.0 ± 0.08	4.5 ± 0.3	14.3 ± 0.9	10.9 ± 0.2	29.5 ± 0.3	5.1 ± 0.2
**2a**	>50 [b]	>50 [b]	>50 [b]	>50 [b]	>50 [b]	>50 [b]
**b**	12.4 ± 0.6	10.4 ± 0.2	15.1 ± 0.4	16.7 ± 0.5	29.1 ± 0.9	13.1 ± 0.2
**1b**	2.2 ± 0.3	5.8 ± 0.5	8.4 ± 0.3	11.4 ± 0.6	25.9 ± 0.4	3.9 ± 0.6
**2b**	>50 [b]	>50 [b]	>50 [b]	>50 [b]	>50 [b]	>50 [b]
**Cisplatin**	1.2 ± 0.3	3.2 ± 0.1	5.2 ± 0.3	8.7 + 0.9	5.7 ± 0.9	4.1 ± 0.5

[a] Cellular viability determined using the MTT assay after 24 h drug exposure time at 37 °C and 72 h recovery in drug-free media. [b] No activity observed in concentration range investigated.

**Table 2:** Antiproliferative activities of prepared compounds towards selected ovarian cancer cell lines.

Compound	IC_50_ (µM) [a]				
	A2780	SKOV3	SW626	A2780Cis	A2780ADR
**a**	8.6 ± 0.2	22.5 ± 0.4	8.4 ± 0.6	n.d. [c]	n.d. [c]
**1a**	1.0 ± 0.08	6.4 ± 0.2	3.8 ± 0.4	1.1 ± 0.05	1.6 ± 0.2
**2a**	>50 [b]	>50 [b]	>50 [b]	n.d. [c]	n.d. [c]
**b**	12.4 ± 0.6	20.3 ± 0.6	6.4 ± 0.5	n.d. [c]	n.d. [c]
**1b**	2.2 ± 0.3	5.1 ± 0.2	2.8 ± 0.4	2.5 ± 0.1	2.8 ± 0.4
**2b**	>50 [b]	>50 [b]	>50 [b]	n.d. [c]	n.d. [c]
**Cisplatin**	1.2 ± 0.3	16.8 ± 0.8	15.7 ±0.8	13.4 ± 0.3	8.9 ± 0.5

[a] Cellular viability determined using the MTT assay after 24 h drug exposure time at 37 °C and 72 h recovery in drug-free media. [b] No activity observed in concentration range investigated. [c] Not determined.

**Table 3:** Cellular accumulation of ruthenium in A2780 cancer cells treated with 1.0 µM of **1a** or **2a** for 24 h at 37 °C. Ruthenium content in cells was measured using HRCS-AAS.

Complex	nmol Ru/mg Protein [a]
**1a**	1.30 ± 0.22
**2a**	1.02 ± 0.15
**Untreated**	0.03 ± 0.09

[a] Protein content determined by the Bradford method.

**Table 4:** Cell cycle analysis of A2780 cancer cells treated with complex 1a (1.0 µM) for 24 h at 37 °C and analyzed by flow cytometry after fixation in ethanol and staining with propidium iodide.

Complex	G1	G2/M	S
**1a**	72.5 ± 0.5	12.6 ± 0.7	14.9 ± 0.6
**Untreated**	62.3 ± 0.3	17.8 ± 0.4	19.9 ± 0.8

**Table 5:** Gated populations for flow cytometric analysis of cellular apoptosis in A2780 cancer cells treated with complex **1a** (1.0 µM) for 24 h at 37 °C. Cells stained with propidium iodide (PI) and Annexin-V-FITC (ANN) without fixation.

Complex	PI−/ANN−	PI+/ANN−	PI−/ANN+	PI+/ANN+
**1a**	76.5 ± 0.9 **	11.2 ± 0.8 **	4.1 ± 0.4 **	8.4 ± 0.7 **
**Untreated**	95.6 ± 0.7	2 ± 1	2 ± 1	0.3 ± 0.2

** *p*-value < 0.01.

## Data Availability

Data is contained within the article or supplementary material.

## Supplementary Materials

The following are available online at https://www.mdpi.com/article/10.3390/cancers13102493/s1, Figure S1: General scheme of A) prepared ligands **a**–**b** and B) their organoruthenium(II) chlorido **1a**–**b** and pta **2a**–**b** complexes. Figure S2: Photos of single crystals for ligand **a** (left) and pta complex **2b** (right). Figure S3: Packing of molecules of ligand **a**. Figure S4: ^1^H NMR spectra of organoruthenium(II) chlorido complex **1a** in 5% (CD_3_)_2_SO/D_2_O containing 140 mM NaCl at the chosen timepoints. Figure S5: ^1^H NMR spectra of organoruthenium(II) chlorido complex **1a** in 5% (CD_3_)_2_SO/D_2_O without NaCl at the chosen timepoints. Figure S6: ^1^H NMR spectra of organoruthenium(II) chlorido complex **1b** in 5% (CD_3_)_2_SO/D_2_O containing 140 mM NaCl at the chosen timepoints. Figure S7: ^1^H NMR spectra of organoruthenium(II) chlorido complex **1b** in 5% (CD_3_)_2_SO/D_2_O without NaCl at the chosen timepoints. Figure S8: ^1^H NMR spectra of organoruthenium(II) pta complex **2a** in 5% (CD_3_)_2_SO/D_2_O containing 140 mM NaCl at the chosen timepoints. Figure S9: ^1^H NMR spectra of organoruthenium(II) pta complex **2a** in 5% (CD_3_)_2_SO/D_2_O without NaCl at the chosen timepoints. Figure S10: 1H NMR spectra of organoruthenium(II) pta complex **2b** in 5% (CD_3_)_2_SO/D_2_O containing 140 mM NaCl at the chosen timepoints. Figure S11: ^1^H NMR spectra of organoruthenium(II) pta complex **2b** in 5% (CD_3_)_2_SO/D_2_O without NaCl at the chosen timepoints. Figure S12: UV-Vis spectra of A) organoruthenium(II) chlorido **1b** and B) pta complex **2b** in PBS, RPMI-1640, FP-RPMI-1640, and human blood plasma recorded immediately after the preparation (black curve) and after 24 h (red curve). Figure S13: ^1^H NMR spectrum of **2a**. Figure S14: ^1^H NMR spectrum of **2b**. Figure S15: Antiproliferative activities of the ruthenium dimer precursor towards A2780 ovarian cancer cells and A549 lung cancer cells. Figure S16: Antiproliferative activities of the prepared compounds towards A2780 ovarian cancer cells. Figure S17: Negative controls for A2780 cancer cells stained with Rhodamine-123 (green) or DAPI (blue). Table S1: Crystallographic data for ligand **a** and complex **2b**.
