# Supplementary material for: Organoruthenium Complexes with Benzo-Fused Pyrithiones Overcome Platinum Resistance in Ovarian Cancer Cells
Source: Cancers (Basel). 2021 May 20;13(10):2493. doi: 10.3390/cancers13102493 (PMC8160969; doi:10.3390/cancers13102493)
Supplement: Supplementary file 1 [file cancers-13-02493-s001.zip › cancers-1199827-supplementary.pdf]

Supplementary Materials: Organoruthenium Complexes with Benzo-fused Pyrithiones Overcome Platinum Resistance in Ovarian Cancer Cells

Jerneja Kladnik, James P. C. Coverdale, Jakob Kljun, Hilke Burmeister, Petra Lippman, Francesca G. Ellis, Alan M. Jones, Ingo Ott, Isolda Romero-Canelón and Iztok Turel

1. Syntheses of organic ligands and organoruthenium(II) complexes

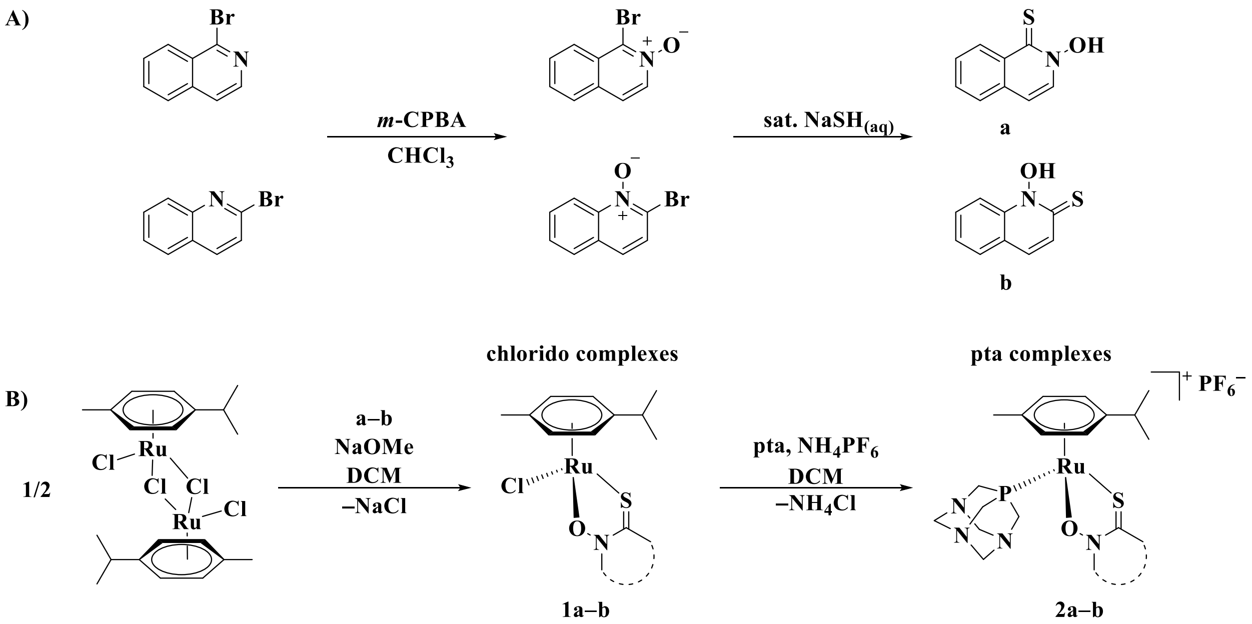

Figure S1. General scheme of (**A**) prepared ligands **a–b** and (**B**) their organoruthenium (II) chlorido **1a–b** and pta **2a–b** complexes.

2. Single crystal X-ray diffraction

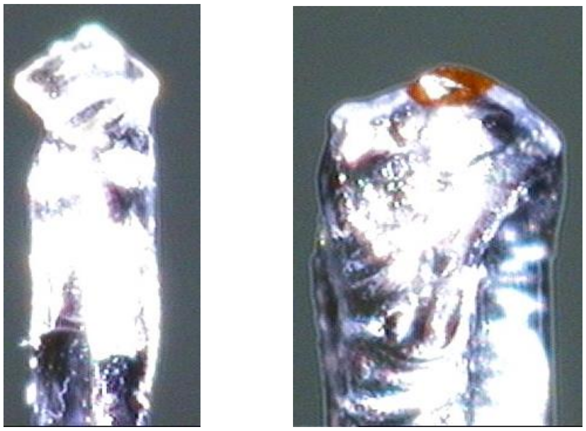

a **2b**

Figure S2. Photos of single crystals for ligand **a** (left) and pta complex **2b** (right).

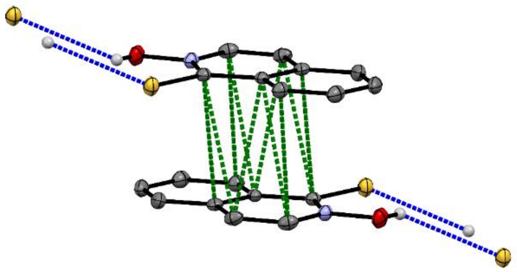

Figure S3. Packing of molecules of ligand **a**. Hydrogen bonds are drawn in blue, while green dotted lines connect carbon atoms with interatomic distance between 3.4 Å and 3.6 Å.

Table S1. Crystallographic data for ligand **a** and complex **2b.**

**Compound**	**a**	**2b**
Empirical formula	C_9H_7NOS	$C_{25}H_{32}F_6N_4OP_2RuS$
Formula weight	177.22	713.61
Temperature/K	150	150
Crystal system	triclinic	monoclinic
Space group	P-1	$P2_1/n$
a/Å	6.8320(5)	9.9036(3)
b/Å	7.2558(5)	17.4561(7)
c/Å	8.6697(7)	16.2456(6)
α/°	71.325(7)	90
β/°	77.248(7)	103.713(4)
γ/°	82.763(6)	90
Volume/Å^3	396.39(5)	2728.45(18)
Z	2	4
ϱ_{calc} g cm^{-3}	1.485	1.737
μ/mm^{-1}	0.349	0.838
F(000)	184.0	1448.0
Crystal size/mm^3	0.2×0.2×0.03	0.1×0.05×0.03
Radiation	MoKα (λ = 0.71073)	MoKα (λ = 0.71073)
Reflections collected	3225	23073
Independent reflections	1814 [R_{int} = 0.0225, R_{sigma} = 0.0399]	6237 [R_{int} = 0.0431, R_{sigma} = 0.0434]
Data/restraints/parameters	1814/0/110	6237/0/364
Goodness-of-fit on F^2	1.081	1.030
Final R indexes [I ≥ 2σ (I)]	R_1 = 0.0390, wR_2 = 0.0896	R_1 = 0.0329, wR_2 = 0.0639
Final R indexes [all data]	R_1 = 0.0516, wR_2 = 0.0973	R_1 = 0.0497, wR_2 = 0.0685
Largest diff. peak/hole / e Å^{-3}	0.39/-0.20	0.75/-0.48

3. NMR stability spectra

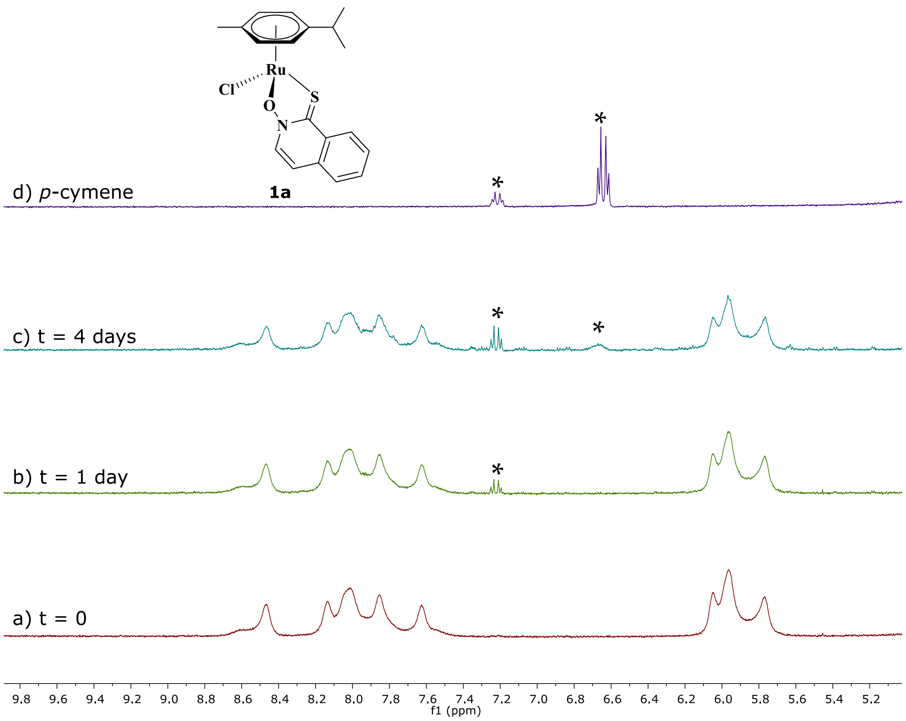

Figure S4. ^{1}H NMR spectra of organoruthenium (II) chlorido complex **1a** in 5% $(CD_3)_2SO/D_2O$ *containing 140 mM NaCl* at the chosen timepoints (the release of *p*-cymene ring is labelled with an asterisk (*)).

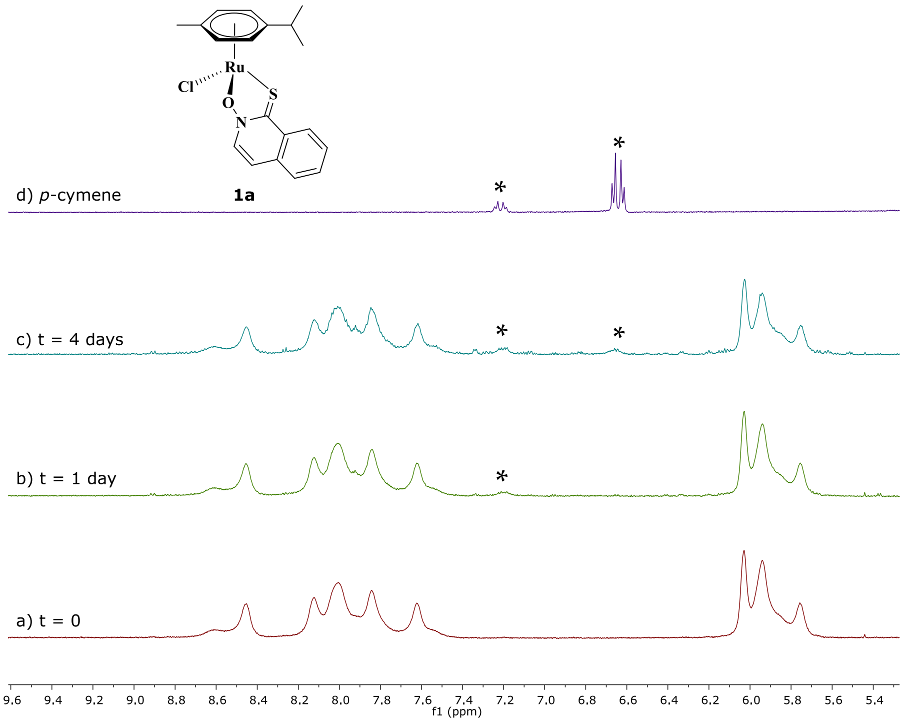

Figure S5. ^{1}H NMR spectra of organoruthenium (II) chlorido complex **1a** in 5% $(CD_3)_2SO/D_2O$ *without NaCl* at the chosen timepoints (the release of *p*-cymene ring is labelled with an asterisk (*)).

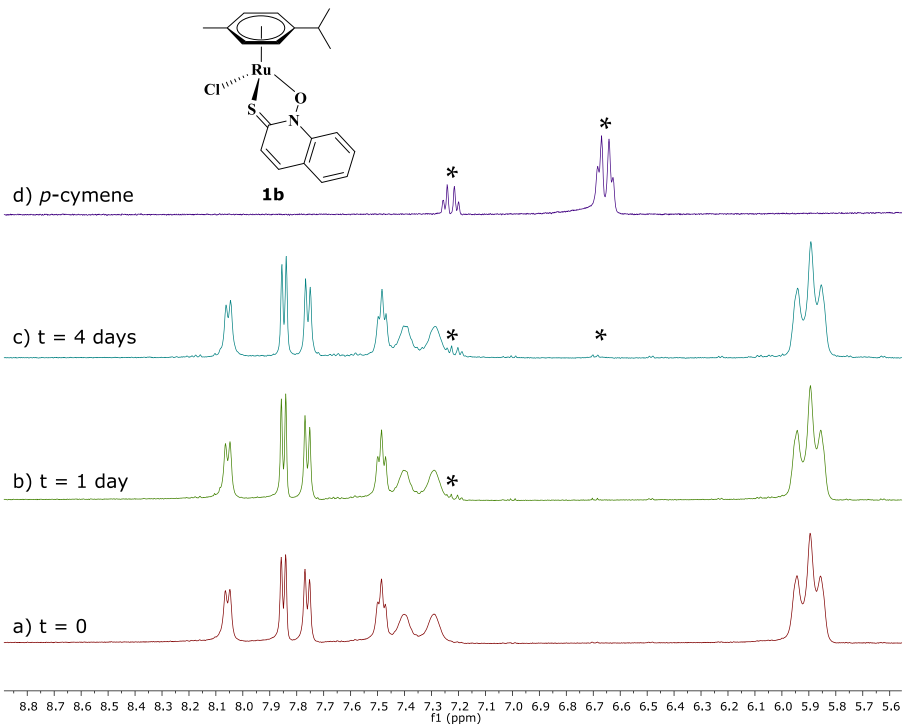

Figure S6. ^{1}H NMR spectra of organoruthenium (II) chlorido complex **1b** in 5% $(CD_3)_2SO/D_2O$ *containing 140 mM NaCl* at the chosen timepoints (the release of *p*-cymene ring is labelled with an asterisk (*)).

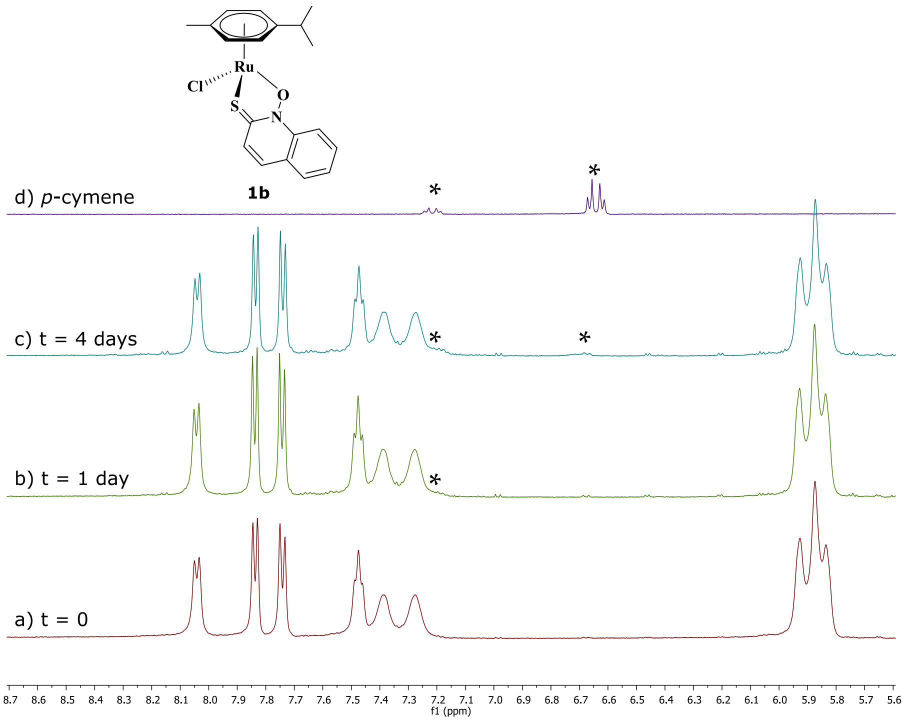

Figure S7. ^{1}H NMR spectra of organoruthenium (II) chlorido complex **1b** in 5% $(CD_3)_2SO/D_2O$ *without NaCl* at the chosen timepoints (the release of *p*-cymene ring is labelled with an asterisk (*)).

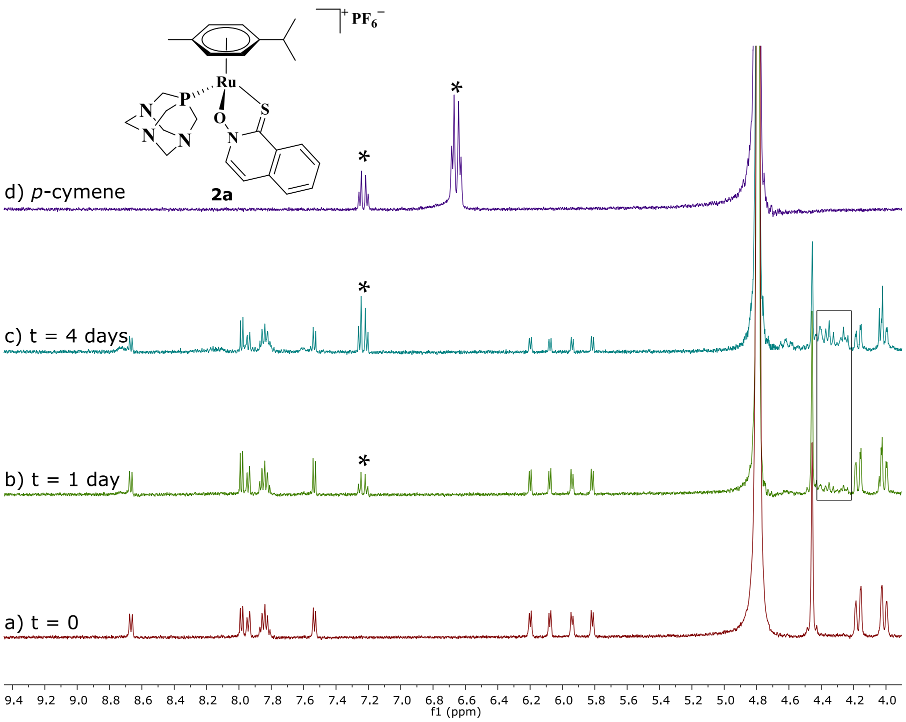

Figure S8. ^{1}H NMR spectra of organoruthenium(II) pta complex **2a** in 5% $(CD_3)_2SO/D_2O$ *containing 140 mM NaCl* at the chosen timepoints (the release of *p*-cymene ring is labelled with an asterisk (*) and possible new phosphine species in a square).

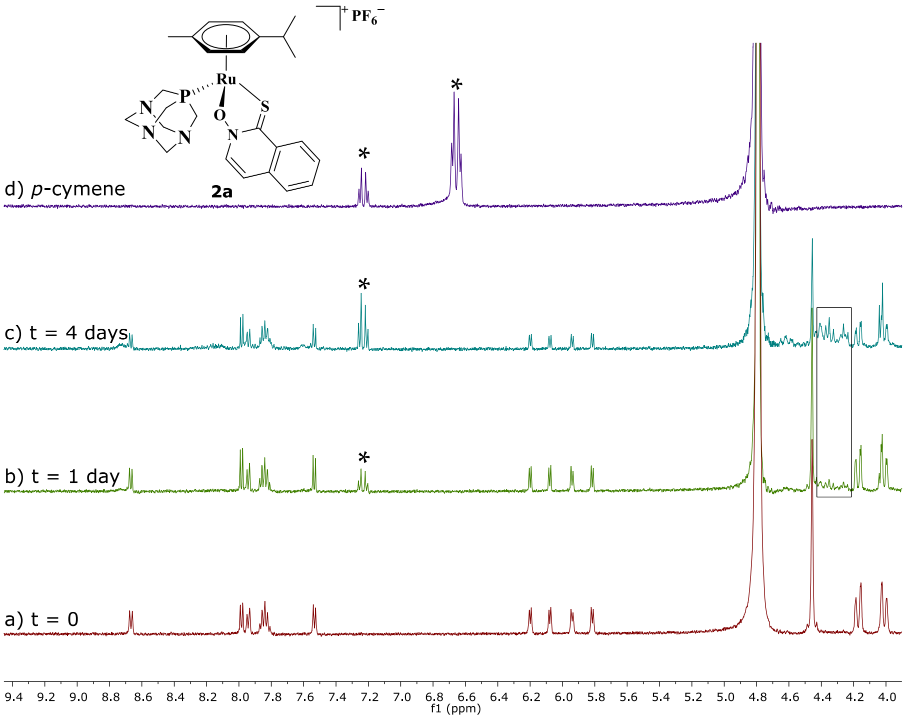

Figure S9. ^{1}H NMR spectra of organoruthenium(II) pta complex **2a** in 5% $(CD_3)_2SO/D_2O$ *without NaCl* at the chosen timepoints (the release of *p*-cymene ring is labelled with an asterisk (*) and possible new phosphine species in a square).

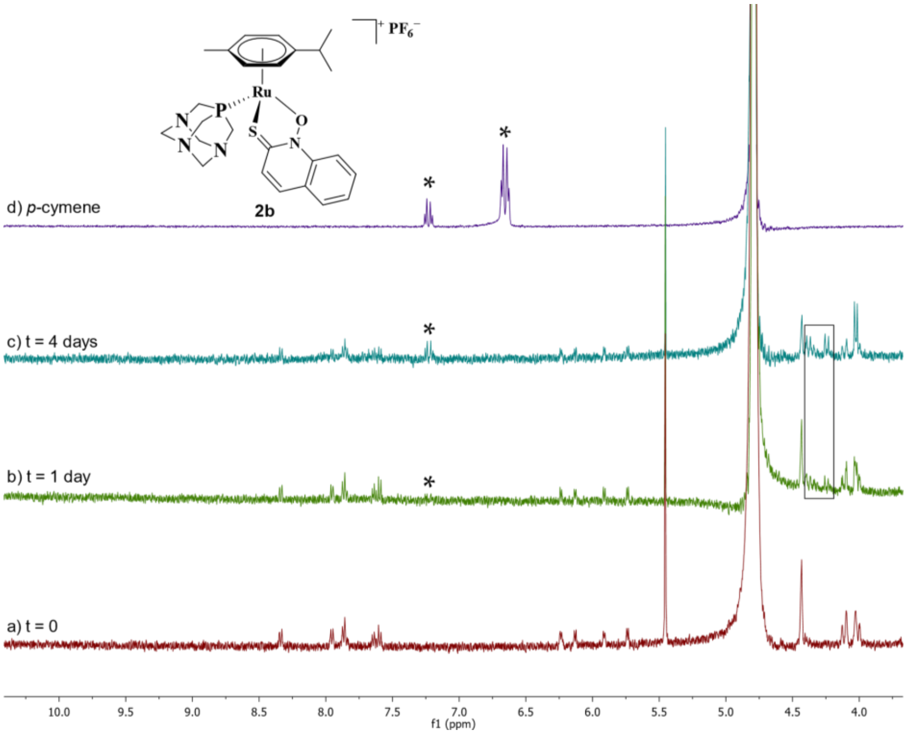

Figure S10. ^{1}H NMR spectra of organoruthenium(II) pta complex **2b** in 5% $(CD_3)_2SO/D_2O$ *containing 140 mM NaCl* at the chosen timepoints (the release of *p*-cymene ring is labelled with an asterisk (*) and possible new phosphine species in a square).

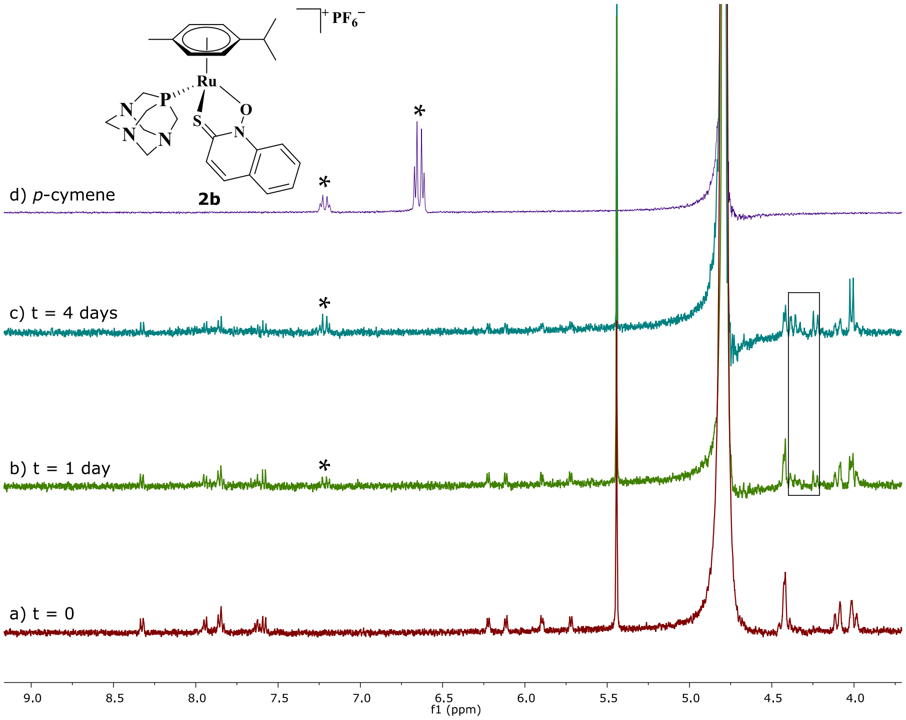

Figure S11. ^{1}H NMR spectra of organoruthenium(II) pta complex **2b** in 5% $(CD_3)_2SO/D_2O$ *without NaCl* at the chosen timepoints (the release of *p*-cymene ring is labelled with an asterisk (*) and possible new phosphine species in a square).

4. UV-Vis stability spectra

A) complex 1b

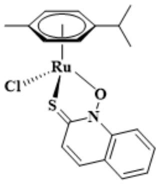

1b

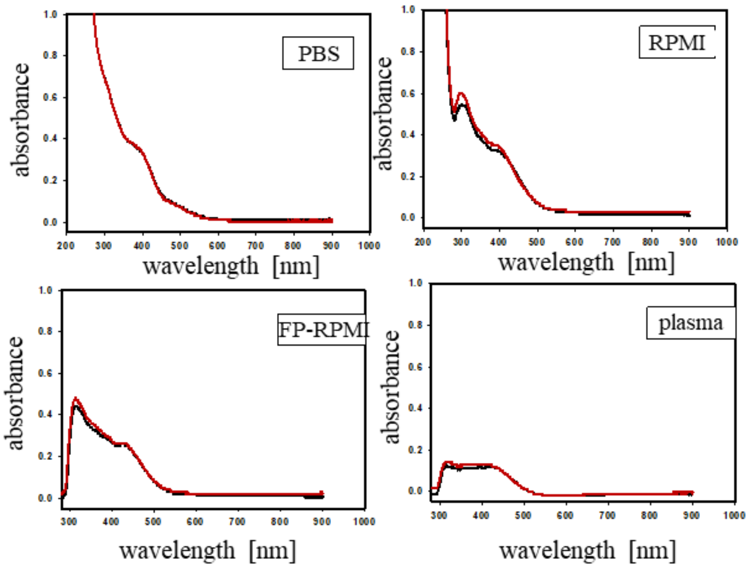

B) complex 2b

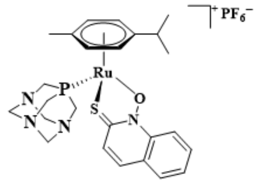

2b

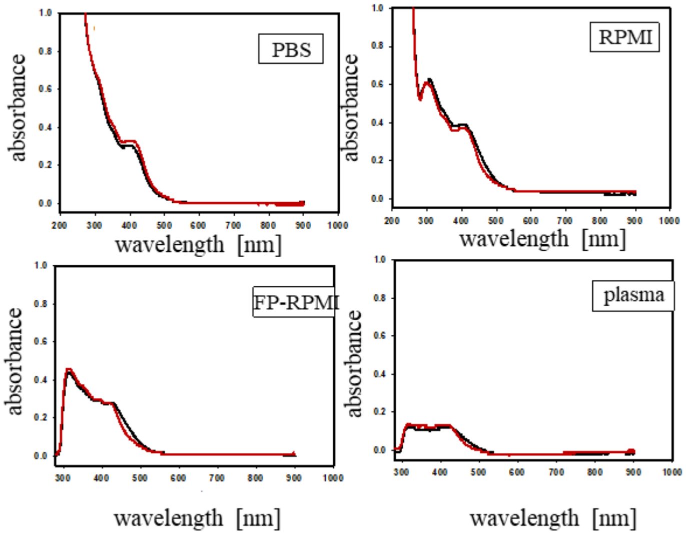

Figure S12. UV-Vis spectra of (**A**) organoruthenium (II) chlorido **1b** and (**B**) pta complex **2b** in PBS, RPMI-1640, FP-RPMI-1640 and human blood plasma recorded immediately after the preparation (black curve) and after 24 h (red curve).

5. NMR spectra of pta complexes 2a–b

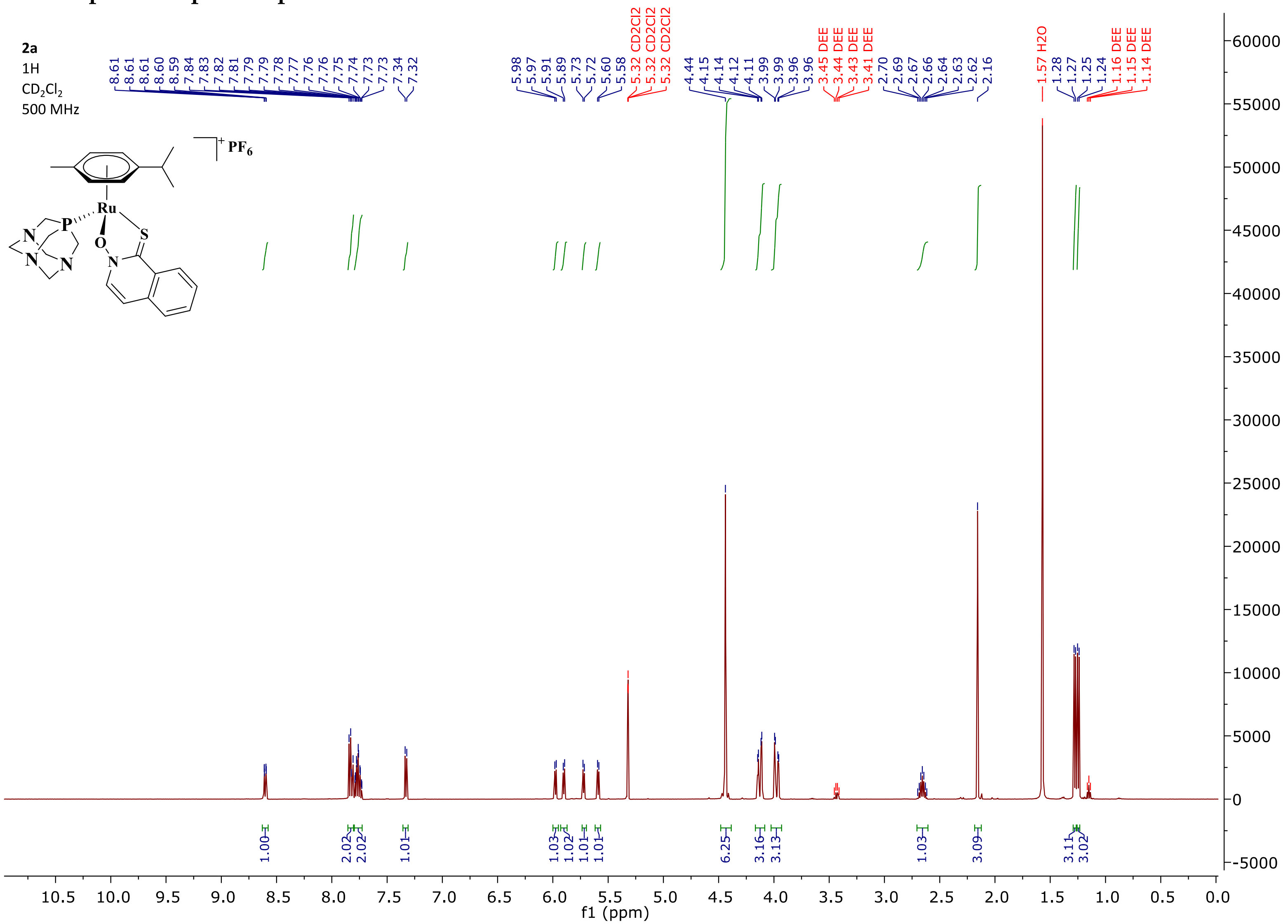

Figure S13. ^{1}H NMR spectrum of **2a**.

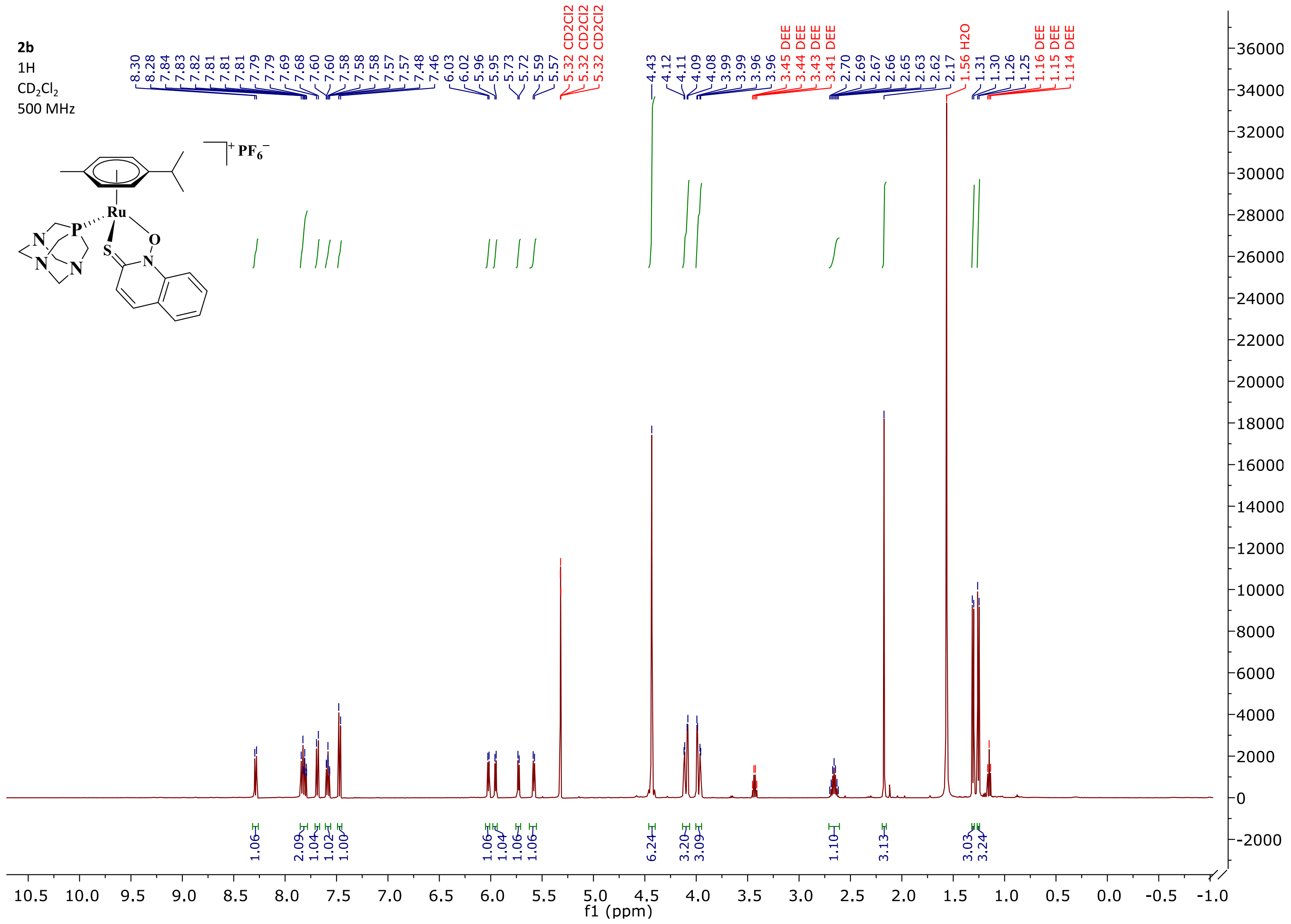

Figure S14. ^{1}H NMR spectrum of **2b**.

6. Biological data

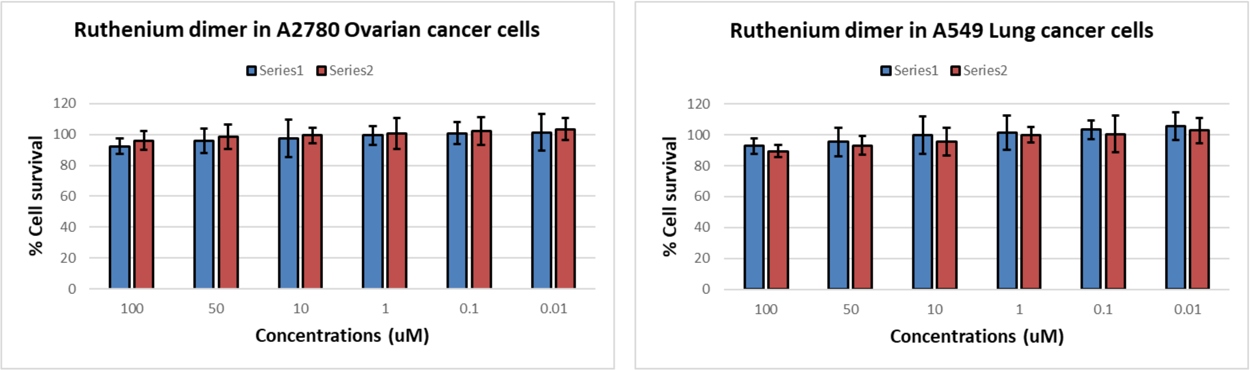

Figure S15: Antiproliferative activities of the ruthenium dimer precursor towards A2780 ovarian cancer cells and A549 lung cancer cells. Cellular viability determined using the MTT assay after 24 h drug exposure time at 37 °C and 72 h recovery in drug-free media. The dimer is inactive under the testing conditions in both cell lines. This is consistent with literature reports [1-5].

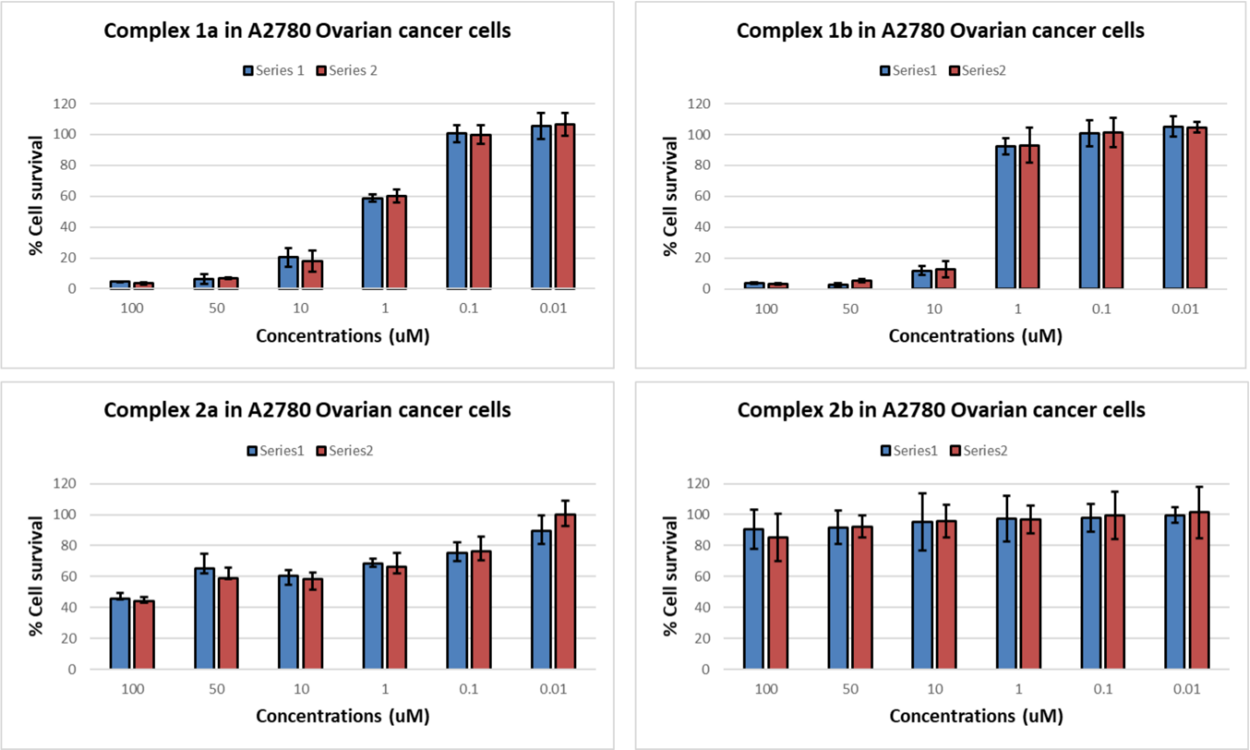

Figure S16: Antiproliferative activities of the prepared compounds towards A2780 ovarian cancer cells. Cellular viability determined using the MTT assay after 24 h drug exposure time at 37 °C and 72 h recovery in drug-free media. The graphs are examples of typical IC_{50} determination, in such case, experiments were carried out in duplicates (series 1 or 2) of triplicates (in each independent series). Sigmoidal curves were fitted from concentration in μM vs. cellular proliferation (%) as above, with inflection points reflecting the IC_{50} values reported.

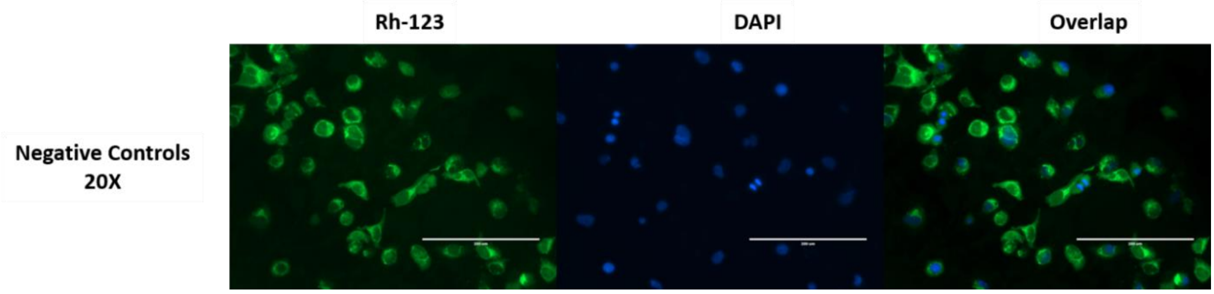

Figure S17: Negative controls for A2780 cancer cells stained with Rhodamine-123 (green) or DAPI (blue). Fluorescence measured using an EVOS fluorescence microscope using Rh-123 (λ_{Ex} 511 nm; λ_{Em} 534 nm) and DAPI (λ_{Ex} 340 nm; λ_{Em} 488 nm).

References

1. Soldevila-Barreda, J.J.; Romero-Canelón, I.; Habtemariam, A.; Sadler, P.J. Transfer hydrogenation catalysis in cells as a new approach to anticancer drug design. *Nat. Commun.* **2015**, *6*, 6582, doi:10.1038/ncomms7582.
2. Namiecińska, E.; Sadowska, B.; Więckowska-Szakiel, M.; Dołęga, A.; Pasternak, B.; Grazul, M.; Budzisz, E. Anticancer and antimicrobial properties of novel η^6-*p*-cymene ruthenium(II) complexes containing a N,S-type ligand, their structural and theoretical characterization. *RSC Adv.* **2019**, *9*, 38629–38645, doi:10.1039/c9ra08736b.
3. Oehninger, L.; Stefanopoulou, M.; Alborzinia, H.; Schur, J.; Ludewig, S.; Namikawa, K.; Muñoz-Castro, A.; Köster, R.W.; Baumann, K.; Wölfl, S.; et al. Evaluation of arene ruthenium(II) *N*-heterocyclic carbene complexes as organometallics interacting with thiol and selenol containing biomolecules. *Dalton Trans.* **2013**, *42*, 1657–1666, doi:10.1039/c2dt32319b.
4. Demoro, B.; de Almeida, R.F.M.; Marques, F.; Matos, C.P.; Otero, L.; Pessoa, J.C.; Santos, I.; Rodriguez, A.; Moreno, V.; Lorenzo, J.; et al. Screening organometallic binuclear thiosemicarbazone ruthenium complexes as potential anti-tumour agents: cytotoxic activity and human serum albumin binding mechanism. *Dalton Trans.* **2013**, *42*, 7131–7146, doi:10.1039/c3dt00028a.
5. Mitra, R.; Das, S.; Shinde, S.V.; Sinha, S.; Somasundaram, K.; Samuelson, A.G. Anticancer activity of hydrogen-bond-stabilized half-sandwich Ru^{II} complexes with heterocycles. *Chem. Eur. J.* **2012**, *18*, 12278–12291, doi:10.1002/chem.201200938.
